# Lipids, Blood Pressure, and Diabetes Mellitus on Risk of Cardiovascular Diseases in East Asians: A Mendelian Randomization Study

**DOI:** 10.1016/j.amjcard.2023.08.007

**Published:** 2023-08-24

**Authors:** Jonathan L. Ciofani, Daniel Han, Usaid K. Allahwala, Benjamin Woolf, Dispender Gill, Ravinay Bhindi

**Affiliations:** a Department of Epidemiology and Biostatistics, School of Public Health, Imperial College London, London, United Kingdom; b Department of Cardiology, Royal North Shore Hospital, Sydney, New South Wales, Australia; c Faculty of Medicine and Health, The University of Sydney, Sydney, New South Wales, Australia; d Medical Research Council Laboratory of Molecular Biology, Cambridge, United Kingdom; e Department of Physiology, Development and Neuroscience, University of Cambridge, Cambridge, United Kingdom; f School of Mathematics and Statistics, University of New South Wales, Sydney, New South Wales, Australia; g Medical Research Council Integrative Epidemiology Unit, University of Bristol, Bristol, United Kingdom; h School of Psychological Science, University of Bristol, Bristol, United Kingdom; i Faculty of Epidemiology and Population Health, London School of Hygiene and Tropical Medicine, London, United Kingdom; j Chief Scientific Advisor Office, Novo Nordisk, Copenhagen, Denmark

**Keywords:** lipids, blood pressure, diabetes mellitus, ischemic heart disease, atrial fibrillation, heart failure, peripheral vascular disease, stroke, mendelian randomisation, genetics

## Abstract

Elevated blood pressure, dyslipidemia, and impaired glycemic control are well-established cardiovascular risk factors in Europeans, but there are comparatively few studies focused on East Asian populations. This study evaluated the potential causal relations between traditional cardiovascular risk factors and disease risk in East Asians through a 2-sample Mendelian randomization approach. We collected summary statistics for blood pressure parameters, lipid subsets, and type 2 diabetes mellitus liability from large genome-wide association study meta-analyses conducted in East Asians and Europeans. These were paired with summary statistics for ischemic heart disease (IHD), ischemic stroke (IS), peripheral vascular disease, heart failure (HF) and atrial fibrillation (AF). We performed univariable Mendelian randomization analyses for each exposure-outcome pair, followed by multivariable analyses for the available lipid subsets. The genetically predicted risk factors associated with IHD and AF were similar between East Asians and Europeans. However, in East Asians only genetically predicted elevated blood pressure was significantly associated with IS (odds ratio 1.05, 95% confidence interval 1.04 to 1.06, p < 0.0001) and HF (odds ratio 1.05, 95% confidence interval 1.04 to 1.06, p < 0.0001), whereas nearly all genetically predicted risk factors were significantly associated with IS and HF in Europeans. In conclusion, this study provides supportive evidence for similar causal relations between traditional cardiovascular risk factors and IHD and AF in both East Asian and European ancestry populations. However, the identified risk factors for IS and HF differed between East Asians and Europeans, potentially highlighting distinct disease etiologies between these populations.

Dyslipidemia, hypertension, and type 2 diabetes mellitus (T2DM) have consistently been identified as common modifiable risk factors for cardiovascular diseases. However, most evidence comes from European ancestry populations,^1–3^ with comparatively little evidence from subjects of East Asian ancestry despite comprising at least one-fifth of the world population. Several observational studies have demonstrated notable epidemiologic differences in the burden of cardiovascular diseases for East Asians compared with Europeans and North Americans.^4–6^ However there remains a relative scarcity of randomized trials performed in East Asian populations to investigate these observational findings. Mendelian randomization (MR) is a research methodology that can test potentially causal relations between cardiovascular risk factors and diseases. A proportion of the phenotype of an individual is determined by genetic polymorphisms that are randomly inherited at birth. The random allocation of phenotype-determining genetic polymorphisms is analogous to assignment to a treatment group in a randomized control trial (Figure 1). MR leverages this random and non-modifiable allocation of genetic variants to help eliminate the risk of bias because of reverse causality and minimize bias from confounding, both of which typically hinder interpretation of nonrandomized observational studies. The present study therefore investigates the potentially causal relations between traditional cardiovascular risk factors and common cardiovascular diseases in East Asian ancestry individuals and compares results with those obtained in European ancestry individuals.

## Methods

The present study uses a 2-sample MR approach. The exposure variables included low-density lipoprotein cholesterol (LDL-c), triglycerides (TGs), high-density lipoprotein cholesterol (HDL-c), systolic blood pressure (SBP), diastolic blood pressure (DBP) and T2DM liability. Ischemic heart disease (IHD), ischemic stroke (IS), peripheral vascular disease (PVD), heart failure (HF), and atrial fibrillation (AF) were used as the outcome variables. Population homogeneity is a requirement for the samples used in a 2-sample MR analysis, and thus both exposure and outcome data were sourced from either East Asian or European ancestry populations, according to the specified ancestry in the analysis. MR makes 3 further core assumptions which are that the genetic variants should be: (1) strongly associated with the exposure; (2) exclusively associated with the outcome through the exposure; and (3) independent of potential confounders. To address the first criterion, uncorrelated genetic variants were selected that were significantly associated with the exposure subsets at p <5 × 10^−8^; and to evaluate the latter 2 criteria, sensitivity analyses were performed as described later.

Data for the present study are publicly available. Ethical approval and consent were obtained by the original studies. Preparation of this manuscript was based on the Strengthening the Reporting of Observational Studies in Epidemiology using Mendelian Randomization Guidelines.^7^

For the East Asian analyses: the data for lipid subsets was extracted from a genome-wide association study (GWAS) meta-analysis performed by the Global Lipids Genetic Consortium for LDL-c, HDL-c and log TG using data from 40 cohorts which included 146,492 East Asian individuals^8^ (summary statistics available at: http://csg.sph.umich.edu/willer/public/glgc-lipids2021/); BP data were extracted from a GWAS meta-analysis of SBP and DBP from 183,785 East Asian individuals, including 125,778 participants from BioBank Japan (BBJ) (summary statistics for significant SNPs are available from the Supplementary Table of the original manuscript)^9^; and T2DM liability data were extracted from a GWAS meta-analysis of 433,540 East Asian ancestry individuals from 23 cohorts, including 191,832 individuals from BBJ (summary statistics available at: https://blog.nus.edu.sg/agen/).^10^

For the European analyses: the LDL-c, HDL-c, and log TG data were similarly extracted from a GWAS meta-analysis performed by the Global Lipids Genetic Consortium using data from up to 146 cohorts and 1,320,016 participants^8^ (summary statistics available at: http://csg.sph.umich.edu/willer/public/glgc-lipids2021/); BP data were extracted from a GWAS meta-analysis of SBP and DBP including up to 1,006,863 European participants (summary statistics for significant SNPs available from the Supplementary Table of the original manuscript)^11^; and T2DM liability data were extracted from a GWAS meta-analysis of DIAGRAM, GERA and UK Biobank including up to 659,316 European ancestry individuals (summary statistics available at: https://www.ebi.ac.uk/gwas/studies/GCST006867).^12^

Single-nucleotide polymorphisms (SNPs) that were associated with each exposure variable at p <5 × 10^−8^ were extracted. Linkage disequilibrium (LD) clumping was performed (*r*^2^ <0.001, 10 megabase distance cutoff, East Asian or European ancestry participants of the 1000 genomes project) and the variants with the smallest p values were selected for further analysis.

GWAS analyses of BBJ data were used for all East Asian outcomes: myocardial infarction (MI),^13^ IS,^13^ peripheral artery disease (PAD),^13^ chronic HF (CHF),^13^ and AF.^14^ For each outcome, this included: 14,992 MI cases and 146,212 controls; 22,664 IS cases and 152,022 controls; 4,112 PAD cases and 173,601 controls; 10,540 CHF cases and 169,186 controls; and 8,180 AF cases and 28,612 controls. Cases were defined by past medical history and textmining of electronic medical records. Data for MI, IS, PAD, and CHF are available from: https://pheweb.jp/pheno/MI.^13^ Data for AF is available from: http://jenger.riken.jp/en/result.^14^ For the purpose of reporting results later, MI is reported as IHD, PAD as PVD, and CHF as HF.

For the European ancestry analyses: IHD data were extracted from a meta-analysis of 48 studies including 60,801 coronary artery disease cases and 123,504 controls, with 77% of participants of European ancestry (summary statistics available at: http://www.cardiogramplusc4d.org/data-downloads/)^15^; IS data were extracted from a metaanalysis including 34,217 IS cases and 406,111 controls of European ancestry (summary statistics available at: https://www.ebi.ac.uk/gwas/studies/GCST006908)^16^; PVD data were extracted from the United Kingdom Biobank GWAS pipeline using the Phenome Scan Analysis Tool, which included 1,456 cases and 461,554 controls (summary statistics available from: https://gwas.mrcieu.ac.uk/datasets/ukb-b-4929/)^17^; HF data were extracted from a GWAS meta-analysis of 26 studies including 47,309 cases and 930,014 controls of European ancestry (summary statistics available at: https://www.ebi.ac.uk/gwas/studies/GCST009541)^18^; and AF data were extracted from a GWAS of 6 contributing studies including 60,620 cases and 970,216 controls of European ancestry (summary statistics available at: https://www.ebi.ac.uk/gwas/studies/GCST006414).^19^

Further details about the genotyping methodology and phenotype definitions can be found in the original GWAS manuscripts (Supplementary Table 9).

**Figure 2:**
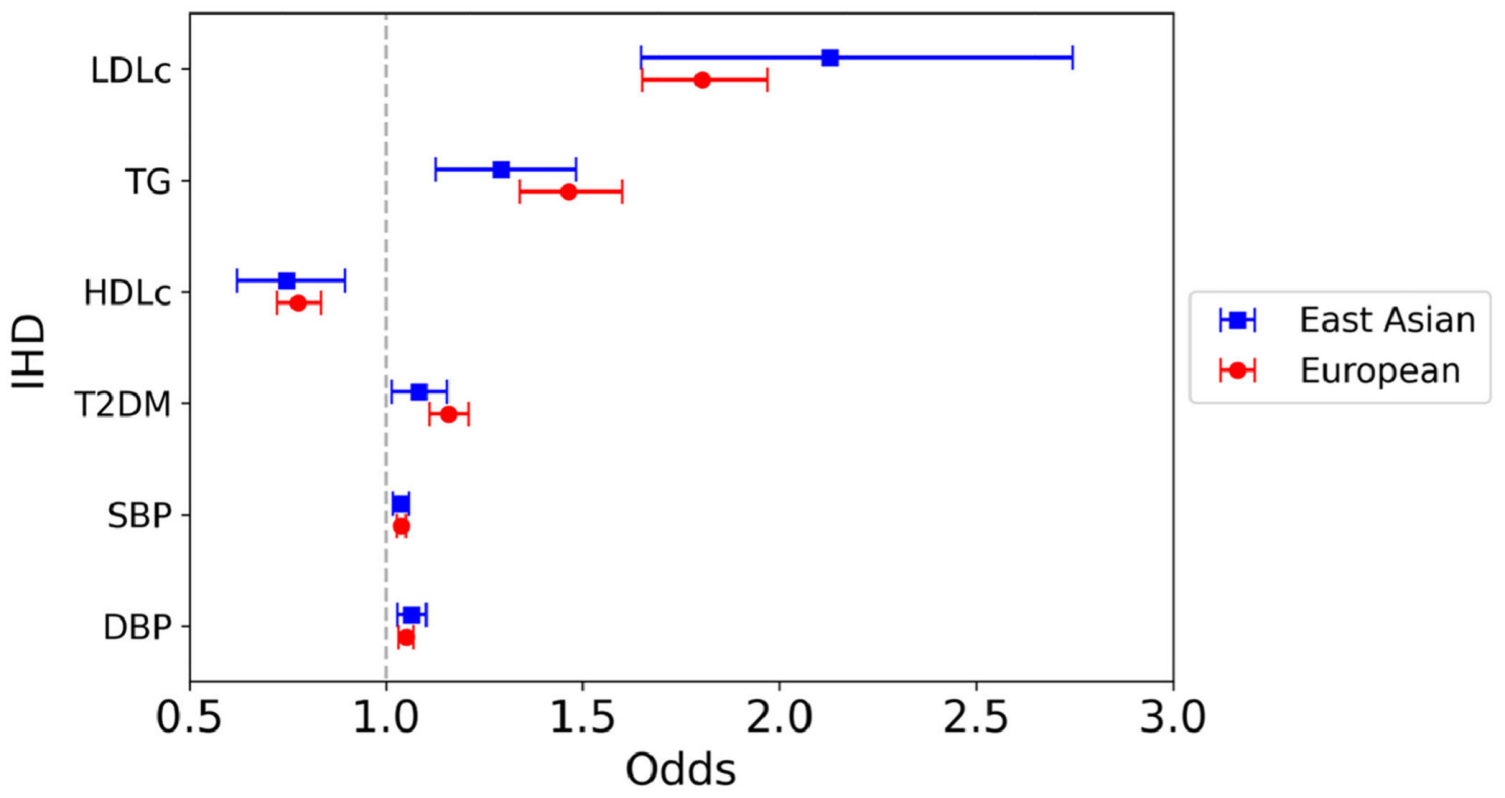
Mendelian randomization inverse variance weighted estimates for the effect of 1 SD increase in genetically predicted elevated LDL-c, TG, and HDL-c, 1 mm Hg increase in SBP, and DBP, and T2DM liability on risk of IHD.

**Figure 3:**
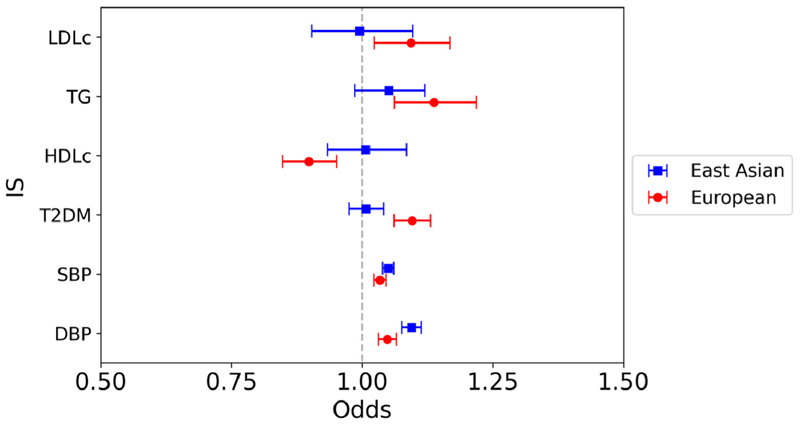
Mendelian randomization inverse variance weighted estimates for the effect of 1 SD increase in genetically predicted elevated LDL-c, TG and HDL-c, 1 mm Hg increase in SBP, and DBP, and T2DM liability on risk of IS.

**Figure 4:**
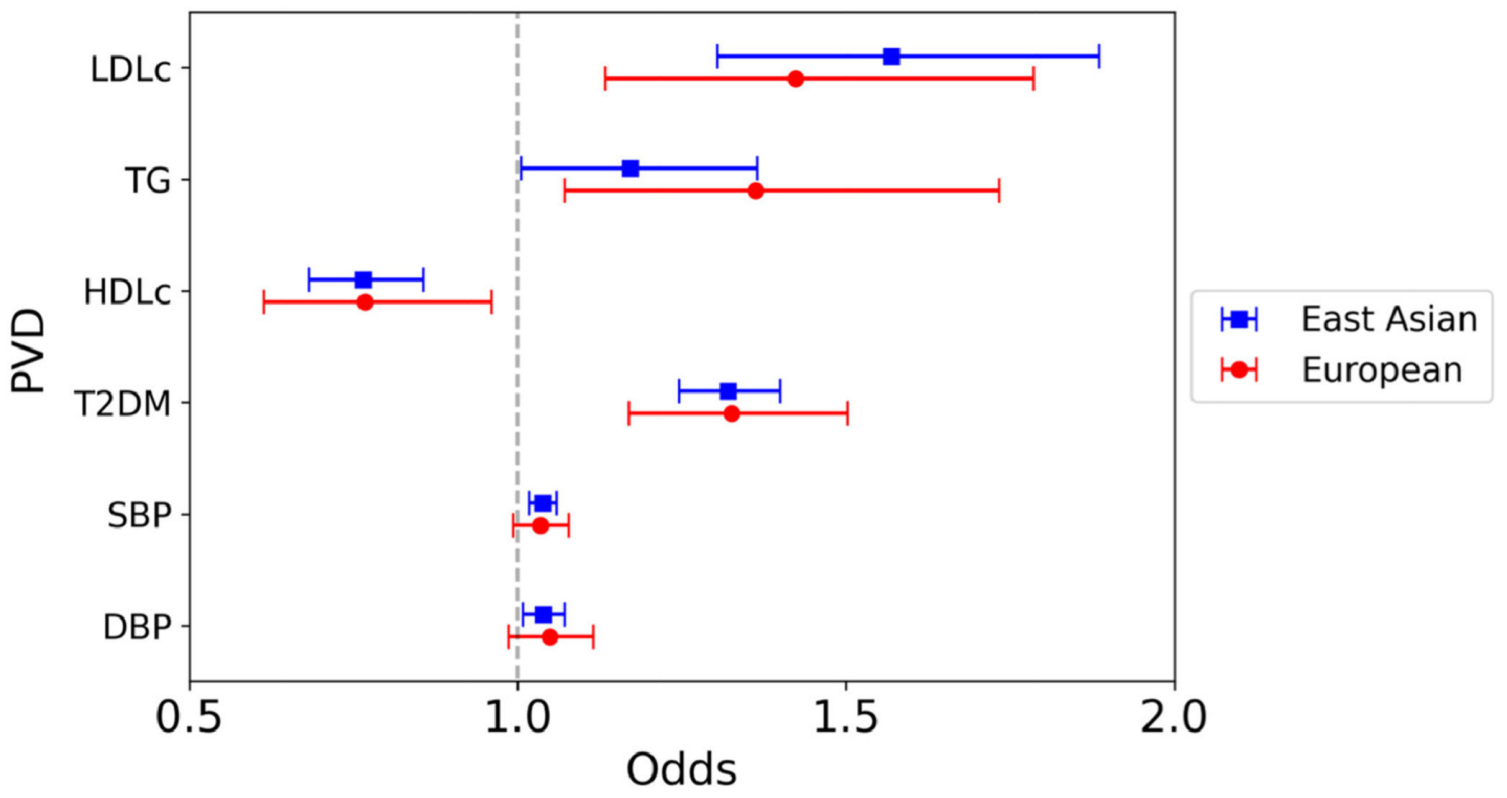
Mendelian randomization inverse variance weighted estimates for the effect of 1 SD increase in genetically predicted elevated LDL-c, TG and HDL-c, 1 mm Hg increase in SBP, and DBP, and T2DM liability on risk of PVD.

**Figure 5:**
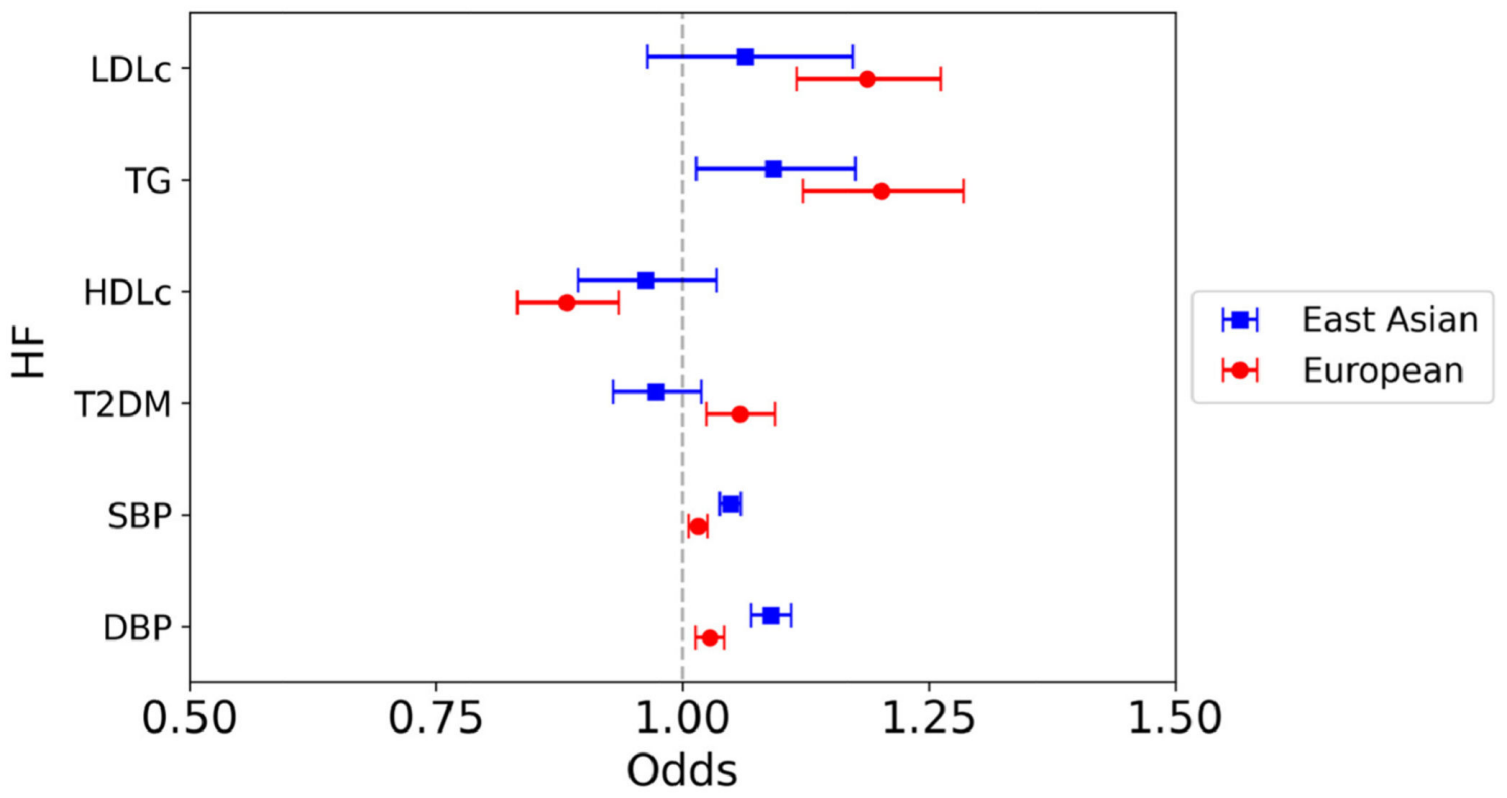
Mendelian randomization inverse variance weighted estimates for the effect of 1 SD increase in genetically predicted elevated LDL-c, TG, and HDL-c, 1 mm Hg increase in SBP and DBP, and T2DM liability on risk of HF.

**Figure 6:**
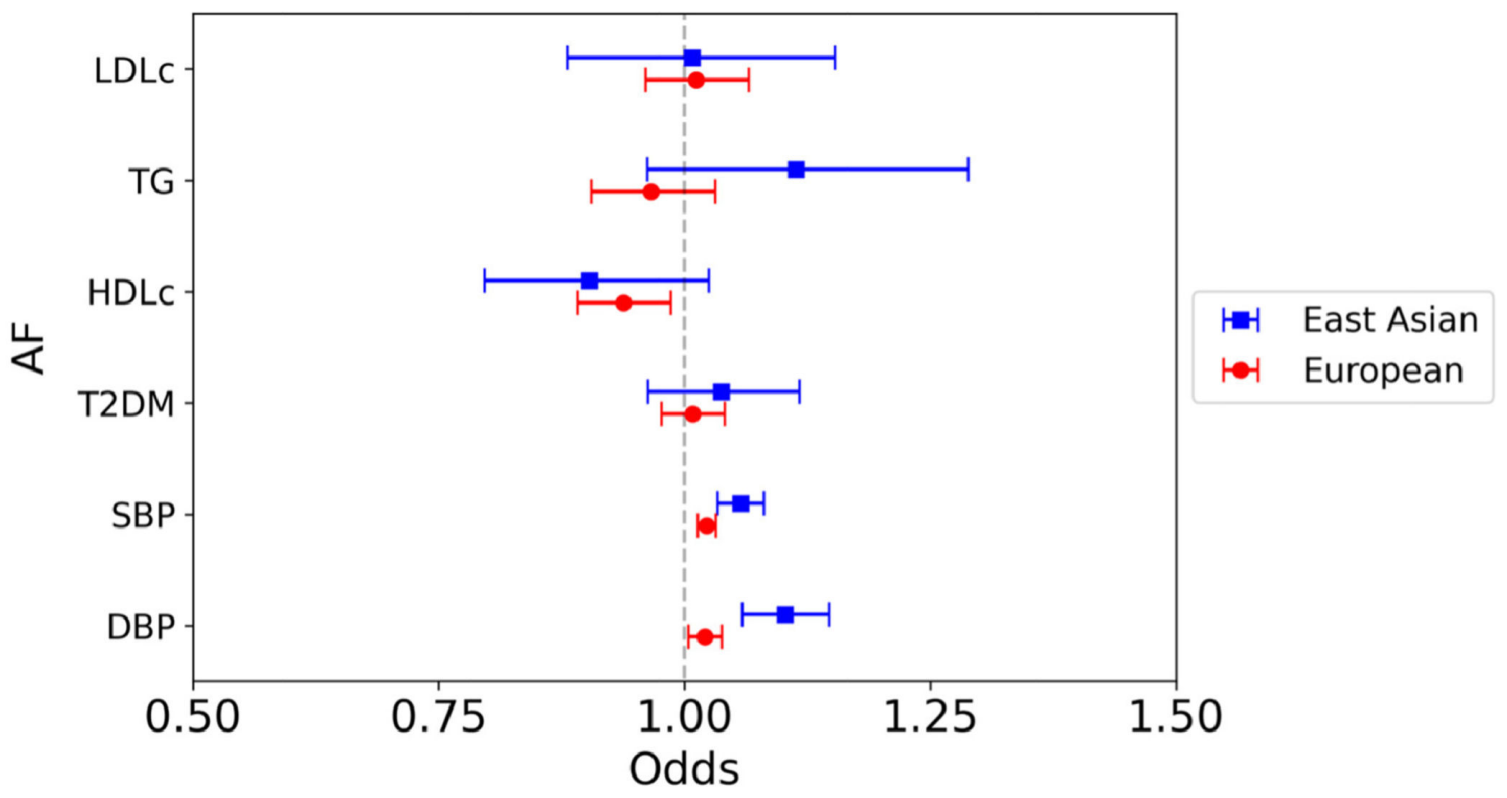
Mendelian randomization inverse variance weighted estimates for the effect of 1 SD increase in genetically predicted elevated LDL-c, TG and HDL-c, 1 mm Hg increase in SBP, and DBP, and T2DM liability on risk of AF.

The primary MR analysis was an inverse variance weighted (IVW) meta-analysis of the Wald ratios for each genetic variant. Results are expressed as odds of the outcome per SD increase in genetically predicted lipid variables and per mm Hg increase in BP variables. A Bonferroni-corrected p value threshold was set at p = 0.0017 for all primary analyses (0.05 / 30, based on 6 exposure and 5 outcome variables) and p = 0.017 for multi-variable analyses if significant on univariable analysis (0.05 / 3, because 3 lipid subsets were included in each model) (Figures 2 to 6, Supplementary Tables 1 to 4).

To evaluate for violation of the main MR assumptions because of horizontal pleiotropy, we performed several sensitivity analyses including weighted-median, weighted-mode, MR-Egger, and MR-RAPS (Supplementary Figure 1, Supplementary Tables 1 and 3). Each method makes different assumptions and thus concordance between methods provides confidence in the conclusion. The weighted-median approach assumes that at least half the instrumental variables are valid. Weighted-mode assumes that the most common causal effect is consistent with the true effect. MR-Egger uses the Instrument Strength Independent of Direct Effect (InSIDE) assumption, which states that the strength of pleiotropic effects from the genetic variants to the outcome is independent of the strength of the association between genetic variants and exposure. Under this assumption, the intercept from MR-Egger analysis estimates the average pleiotropic effect of the genetic variants (Supplementary Table 5). MR-Egger typically has lower power compared with IVW. MR-RAPS is robust to idiosyncratic and systematic pleiotropy, and weak instrument bias. Cochran’s Q statistic was used to test for genetic variant heterogeneity (Supplementary Table 6). Leave-one-out analyses were performed for each exposure-outcome pair (Supplementary Figures 2 and 3). Scatter and funnel plots are presented in Supplementary Figures 4 to 7.

Multivariable MR was subsequently performed to assess whether the effects of the lipid subsets on the outcome were independent of each other (Supplementary Tables 2 and 4, Table 1). Only SNPs that were available in all lipid subsets and outcome summary statistic datasets were considered. Of these, variants associated with at least one lipid trait at p <5 × 10^−8^ were extracted, clumped after ordering from lowest to highest p value of association with any trait (*r*^2^ <0.001, 10 megabase distance cutoff, East Asian, or European ancestry participants of the 1,000 genomes project), and harmonized, before multivariable IVW MR was performed.

Statistical analyses were performed using R version 1.4.1106 with the TwoSampleMR package (The R Foundation for Statistical Computing, Vienna, Austria).

## Results

In the main IVW analyses, a significant association was observed between nearly all traditional cardiovascular risk factors and IHD in East Asians (LDL-c: odds ratio [OR] 2.13, 95% confidence interval [CI] 1.65 to 2.75, p <0.0001; TG: OR 1.29, 95% CI 1.13 to 1.48, p = 0.00026; HDL-c: OR 0.75, 95% CI 0.62 to 0.90, p = 0.0016; SBP: OR 1.04, 95% CI 1.02 to 1.06, p = 0.00032; DBP: OR 1.07, 95% CI 1.03 to 1.10, p = 0.00030) (Figure 2). The association between T2DM and IHD did not reach Bonferroni-corrected statistical significance in East Asians on IVW analysis (OR 1.08, 95% CI 1.02 to 1.16, p = 0.016). In the European analyses, all genetically predicted risk factors, including T2DM, were significantly associated with IHD risk. For both the East Asian and European analyses, multivariable MR accounting for all 3 lipid subsets in the same model demonstrated a similar association between genetically predicted higher LDL-c and lower HDL-c with increased risk of IHD, but there was no strong association between genetically predicted TG and IHD risk in the East Asian analysis (Table 1).

In East Asians, a significant association was observed between the genetically predicted BP parameters and IS on univariable IVW analysis (SBP: OR 1.05, 95% CI 1.04 to 1.06, p <0.0001; DBP: OR 1.09, 95% CI 1.08 to 1.11, p <0.0001), but there was no association between any of the genetically predicted lipid parameters or T2DM on IS risk (Figure 3). This is in contrast to the European analysis, in which significant associations were identified between all risk factor variables, except LDL-c and IS.

Significant associations were observed between genetically predicted LDL-c, HDL-c, T2DM liability, and SBP with risk of PVD (LDL-c: OR 1.57, 95% CI 1.30 to 1.89, p <0.0001; HDL-c: OR 0.76, 95% CI 0.68 to 0.86, p <0.0001; T2DM: OR 1.32, 95% CI 1.25 to 1.40, p <0.0001; SBP: OR 1.04, 95% CI 1.02 to 1.06, p = 0.00027), but not genetically predicted TG or DBP with PVD risk (TG: OR 1.17, 95% CI 1.01 to 1.36, p = 0.043; DBP: OR 1.04, 95% CI 1.01 to 1.07, p = 0.013) (Figure 4). On multivariable MR, there remained a significant association between both LDL-c and HDL-c with PVD. In contrast, for the European analysis only genetically predicted T2DM liability was significantly associated with increased PVD risk on univariable analysis.

Similar to the results for IS, in East Asians a significant association was observed between the genetically predicted BP parameters and HF on univariable IVW analysis (SBP: OR 1.05, 95% CI 1.04 to 1.06, p <0.0001; DBP: OR 1.09, 95% CI 1.07 to 1.11, p <0.0001), but no association between any of the genetically predicted lipid subsets or T2DM liability on HF risk (Figure 5). This contrasts the European analysis, in which significant associations were identified between all analyzed risk factors and HF.

In East Asians, genetically predicted higher BP was associated with increased risk of AF (SBP: OR 1.06, 95% CI 1.03 to 1.08; DBP: OR 1.10, 95% CI 1.06 to 1.15), with no association between any of the genetically predicted lipid subsets or T2DM liability on AF (Figure 6). In the European analysis, genetically predicted higher SBP was associated with increased risk of AF, but DBP, the lipid subsets, and T2DM liability were not.

## Discussion

Blood pressure, lipid levels and T2DM are traditionally considered to be modifiable risks influenced by both genetic and lifestyle factors. The present study leveraged large-scale GWAS data and the MR approach to evaluate the association between traditional cardiovascular risk factors and diseases in East Asian ancestry individuals. We found similar risk factor profiles for IHD and AF between East Asian and European ancestry populations. However, for East Asians, in contrast to Europeans, only genetically predicted elevated BP was significantly associated with IS and HF.

The increased risk of cardiovascular diseases because of dyslipidemia, hypertension, and T2DM has been established in European populations through large observational and randomized trials^1–3^ and extrapolated to non-Europeans in clinical practice. The present study supports this approach for IHD and AF, demonstrating largely concordant findings between East Asian and European ancestry populations for the association between traditional cardiovascular risk factors and these outcomes. However, the present study provided evidence for a causal role of genetically predicted elevated BP, but not dyslipidemia or T2DM, as risk factors for IS and HF in East Asians. Previous studies in East Asians have consistently demonstrated that elevated BP contributes to a substantially higher population-attributable risk toward stroke compared with dyslipidemia and diabetes mellitus.^20^ Existing evidence has additionally demonstrated that East Asians are more frequently diagnosed with masked hypertension, and East Asian populations may experience a higher morning BP surge than Europeans which is predictive of stroke risk.^21^ Indeed, the relatively increased susceptibility of East Asians to elevated BP was recognized by Kario et al^22^ in a targeted consensus statement on hypertension management specifically for Asian populations.

The finding that the lipid subsets were significantly associated with IHD and PVD but not IS, HF, or AF in East Asians was unexpected in the context of the results from European ancestry individuals. This may be related to insufficient statistical power, although comparable findings have previously been reported. The Management of Elevated Cholesterol in the Primary Prevention Group of Adult Japanese (MEGA) trial, which Randomized 7,832 Japanese patients to pravastatin plus diet versus diet alone, showed a significant reduction in risk of MI and overall coronary heart disease but no significant difference in risk of IS in the pravastatin group.^23^ In the more recent but smaller Japan Statin Treatment Against Recurrent Stroke (J-STARS) trial, in which 1,578 Japanese patients with previous noncardioembolic IS and hypercholesterolemia were randomized to pravastatin or control, the pravastatin group experien_ced lower_ rates of atherothrombotic infarct but the overall rate of stroke or transient ischemic attack was not significantly different from the control group.^24^ Neither study reported outcome rates of PVD, HF, or AF. The inverse association between genetically predicted HDL-c and nearly all the outcomes in the European IVW analyses is also interesting in light of recent studies which have questioned the causal association between HDL-c and cardiovascular events.^25,26^ This may reflect the larger sample size in the present study compared with previous analyses. Notably, however, these results were not consistently supported by the sensitivity analyses, which is similar to the report by Burgess and Davey Smith^27^ that demonstrated a significant association in MR analysis between HDL-c and coronary artery disease on IVW but not sensitivity analyses.

Genetic ancestry has previously been shown to modulate the effect of T2DM on risk of diabetic complications. The present study showed an association between genetically predicted T2DM and elevated risk of PVD in East Asians and a trend toward association with IHD that did not reach significance after Bonferroni-correction for multiple testing. Previous studies on the relation between T2DM and cardiovascular diseases in East Asians have not been consistent in their findings. A multiethnic study of 62,432 participants in North America found lower risk of incident MI, congestive HF, stroke and non-traumatic lower extremity amputation, and higher risk of end-stage renal disease in Asian compared with European ancestry individuals.^28^ In contrast, the Action in Diabetes and Vascular Disease study found that Asian participants with T2DM had higher incidence of stroke and nephropathy, and lower incidence of PVD and coronary disease compared with their Eastern European and Australian counterparts.^29^ Notably, neither study was specific to East Asians, and it is well-described that within Asia there is heterogeneity regarding cardiovascular disease epidemiology.^4^

There are several important limitations. Statistical power was limited particularly for the PVD outcome dataset in Europeans, for which there were only 1,456 cases, and consequently the CIs were unsurprisingly wide (Supplementary Table 8). Furthermore, because several of the outcome variables were extracted from biobank data, it is possible that there were inaccuracies in the attribution of participants as a case versus control. Moreover, the outcome definitions between East Asian and European analyses were not identical, as the analyses were limited by the availability of large suitable GWAS datasets. IHD, for example, was defined for East Asians as MI (BBJ) whereas the European dataset included MI, acute coronary syndrome, chronic stable angina, and coronary stenosis >50% (CARDIoGRAM-plusC4D). Importantly, this assumes shared pathogenesis for the spectrum of IHD from mild coronary artery disease to MI. Despite this, the effect of each exposure variable on risk of IHD was comparable between East Asians and Europeans. Furthermore, HF and IS are heterogeneous diseases. IS, for example, has several subtypes with different etiologies that may vary between ancestry groups and this may contribute to the differing risk factor profiles between ancestry groups. Additionally, while there is some overlap between several of the exposure and outcome datasets, the use of strong instruments, achieved by only using SNPs associated with the exposure at p <5 × 10^−8^ with associated high F-statistics (Supplementary Table 7), is expected to mitigate the potential risk of bias. Although there was evidence of significant heterogeneity in many of the analyses, determined by evaluation of the Q statistic, the concordance between the main IVW and sensitivity analyses suggests that bias is less likely to have substantially impacted the results.

Overall, this study used large-scale GWAS data of East Asian individuals to evaluate the relation between traditional cardiovascular risk factors and diseases in a population that is understudied relative to the proportion of the global population. This analysis demonstrated a similar risk factor profile for IHD and AF between East Asians and Europeans but suggested that elevated BP may be the more pertinent risk factor for IS and HF in East Asians. This study presents an important complementary research approach that may better inform further investigation into population-level cardiovascular disease prevention and treatment strategies in East Asian populations.

## Supplementary Material

Supplementary material associated with this article can be found in the online version at https://doi.org/10.1016/j.amjcard.2023.08.007.

Supplementary Figures 1 - 7

Supplementary Table 8

## Figures and Tables

**Figure 1:**
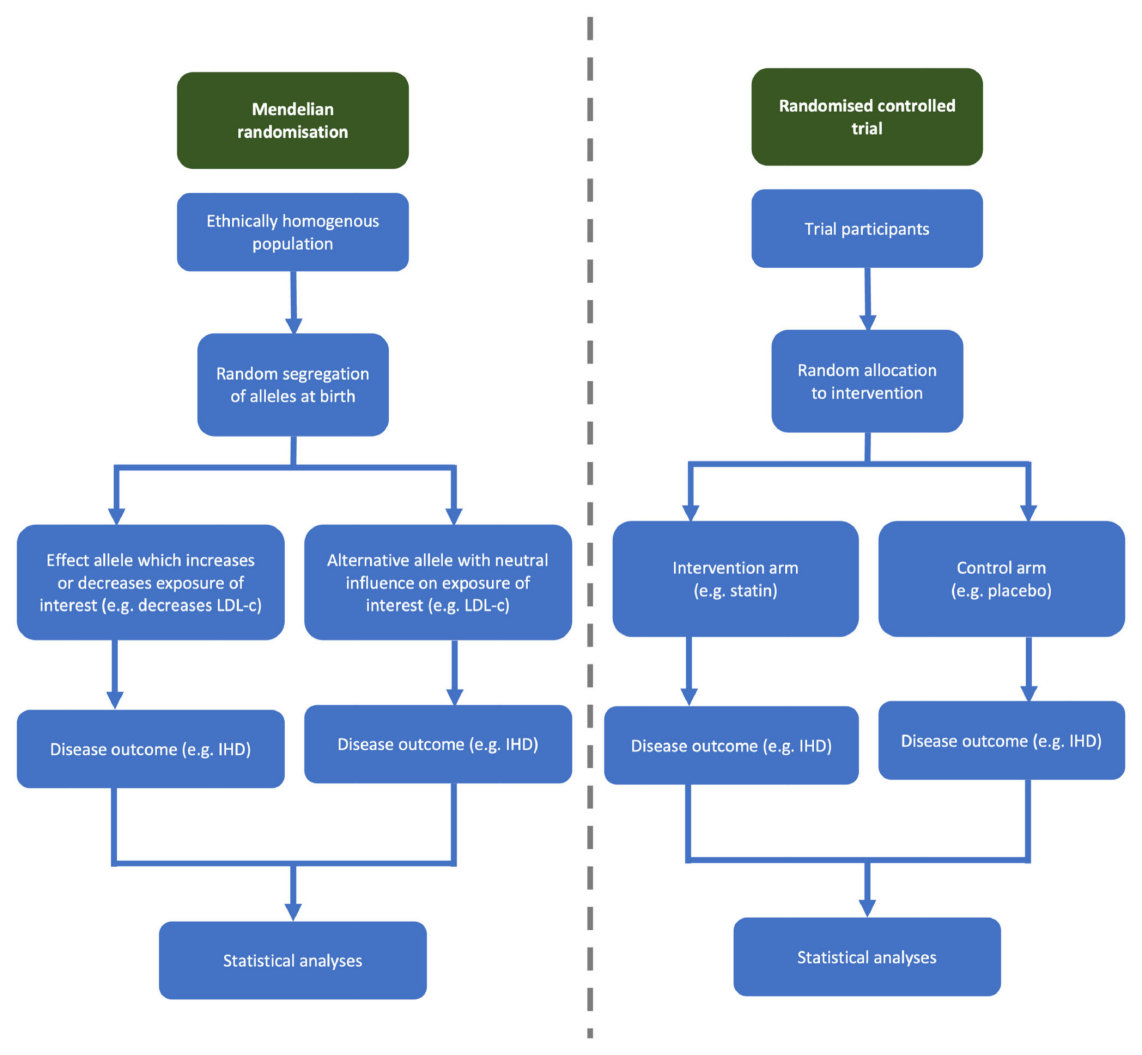
Overview of comparison between a Mendelian randomization study and a randomized controlled trial.

**Table 1:** Multivariable mendelian randomisation estimates for the direct effect of one standard deviation increase in genetically predicted elevated low-density lipoprotein cholesterol, triglycerides and high-density lipoprotein cholesterol respectively, accounting for the other lipid subsets

	Ischemic heart disease Odds (95% CI)	Ischemic stroke Odds (95% CI)	Peripheral vascular disease Odds (95% CI)	Heart failure Odds (95% CI)	Atrial fibrillation Odds (95% CI)
East Asian					
Low-density lipoprotein cholesterol	**2.06 (1.71-2.48)**	0.94 (0.80-1.13)	**2.28 (1.75-2.96)**	1.17 (0.98-1.40)	1.13 (0.77-1.66)
Triglycerides	1.21 (0.95-1.55)	1.40 (1.12-1.77)	0.81 (0.61-1.08)	1.01 (0.79-1.28)	1.37 (0.94-2.00)
High-density lipoprotein cholesterol	**0.69 (0.56-0.86)**	1.09 (0.93-1.29)	**0.44 (0.33-0.58)**	0.83 (0.70-0.99)	0.76 (0.54-1.06)
European					
Low-density lipoprotein cholesterol	**2.01 (1.75-2.31)**	**1.32 (1.07-1.63)**	2.40 (1.24-4.63)	**1.34 (1.18-1.52)**	0.95 (0.79-1.15)
Triglycerides	**1.27 (1.05-1.52)**	1.15 (0.88-1.51)	1.31 (0.64-2.69)	1.21 (0.99-1.46)	0.69 (0.54-0.87)
High-density lipoprotein cholesterol	**0.78 (0.67-0.92)**	0.86 (0.68-1.09)	0.66 (0.41-1.07)	**0.66 (0.56-0.78)**	0.62 (0.50-0.77)
