# Supplementary material for: Lipids, Blood Pressure, and Diabetes Mellitus on Risk of Cardiovascular Diseases in East Asians: A Mendelian Randomization Study
Source: Am J Cardiol. Author manuscript; Available in PMC 2023 Sep 15. (PMC7615095; doi:10.1016/j.amjcard.2023.08.007)
Supplement: Supplementary Figures 1 - 7 [file EMS187425-supplement-Supplementary_Figures_1___7.docx]

**Supplementary Figures**

**A**


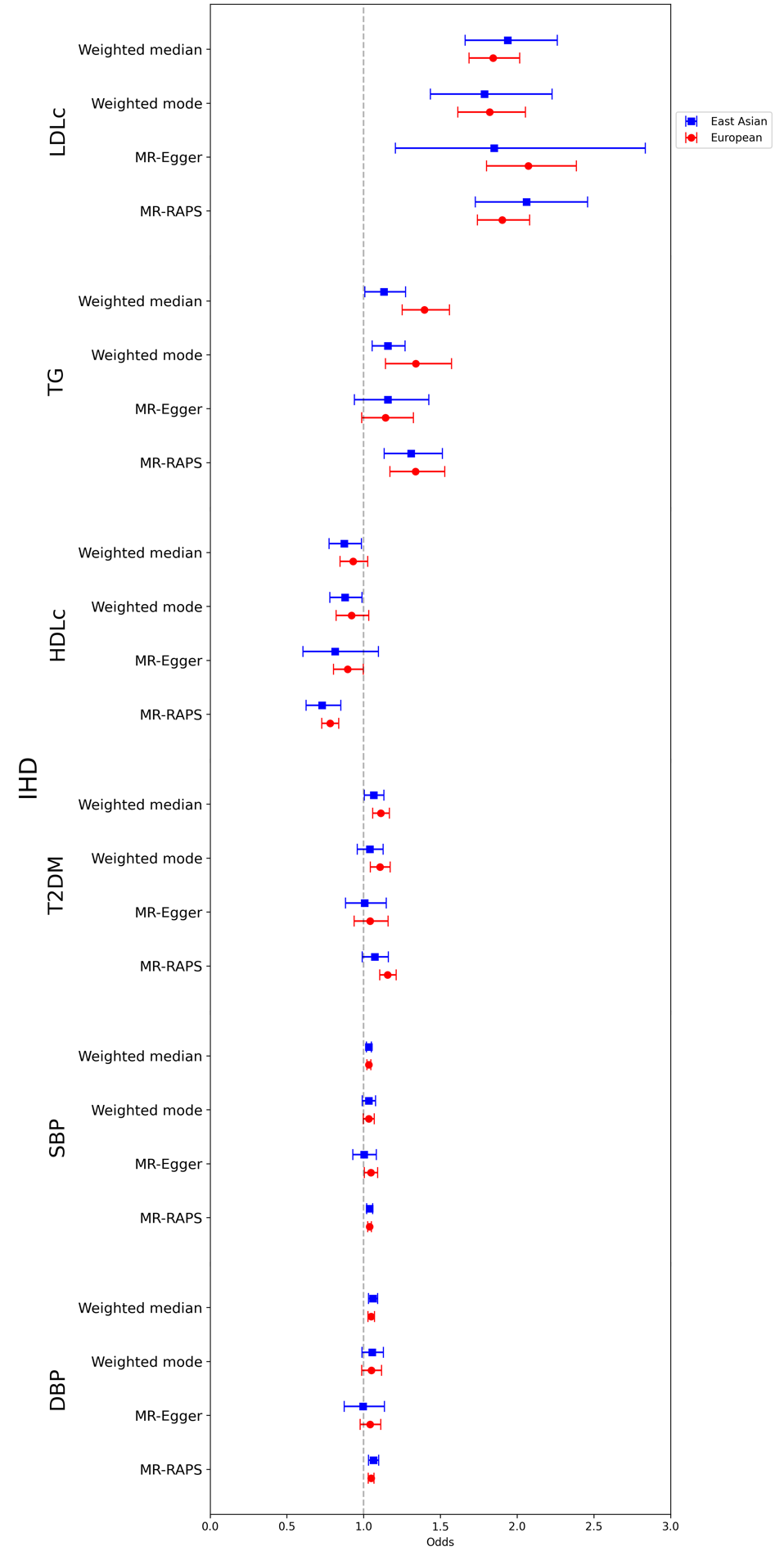


**B**


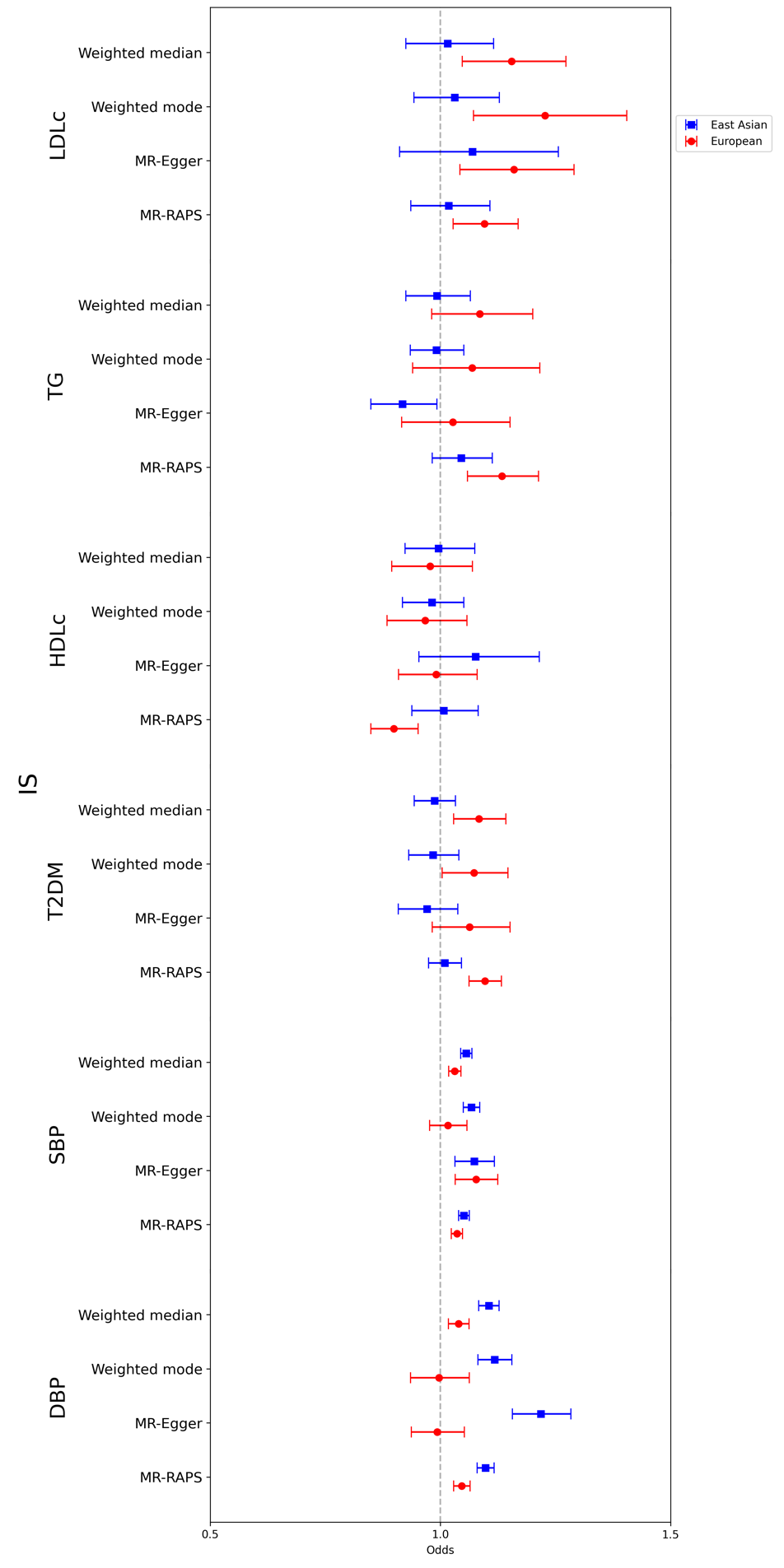


**C**


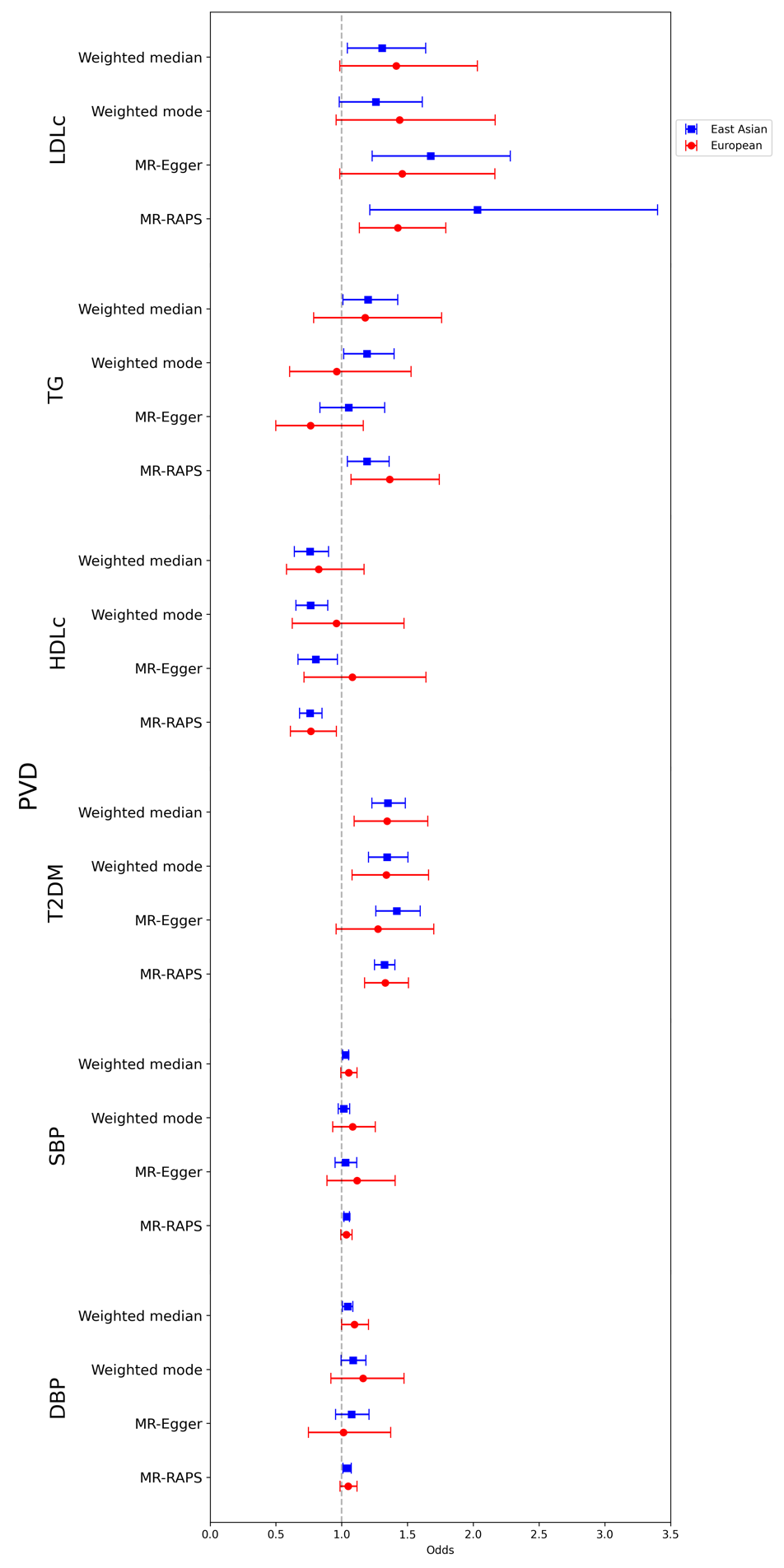


**D**


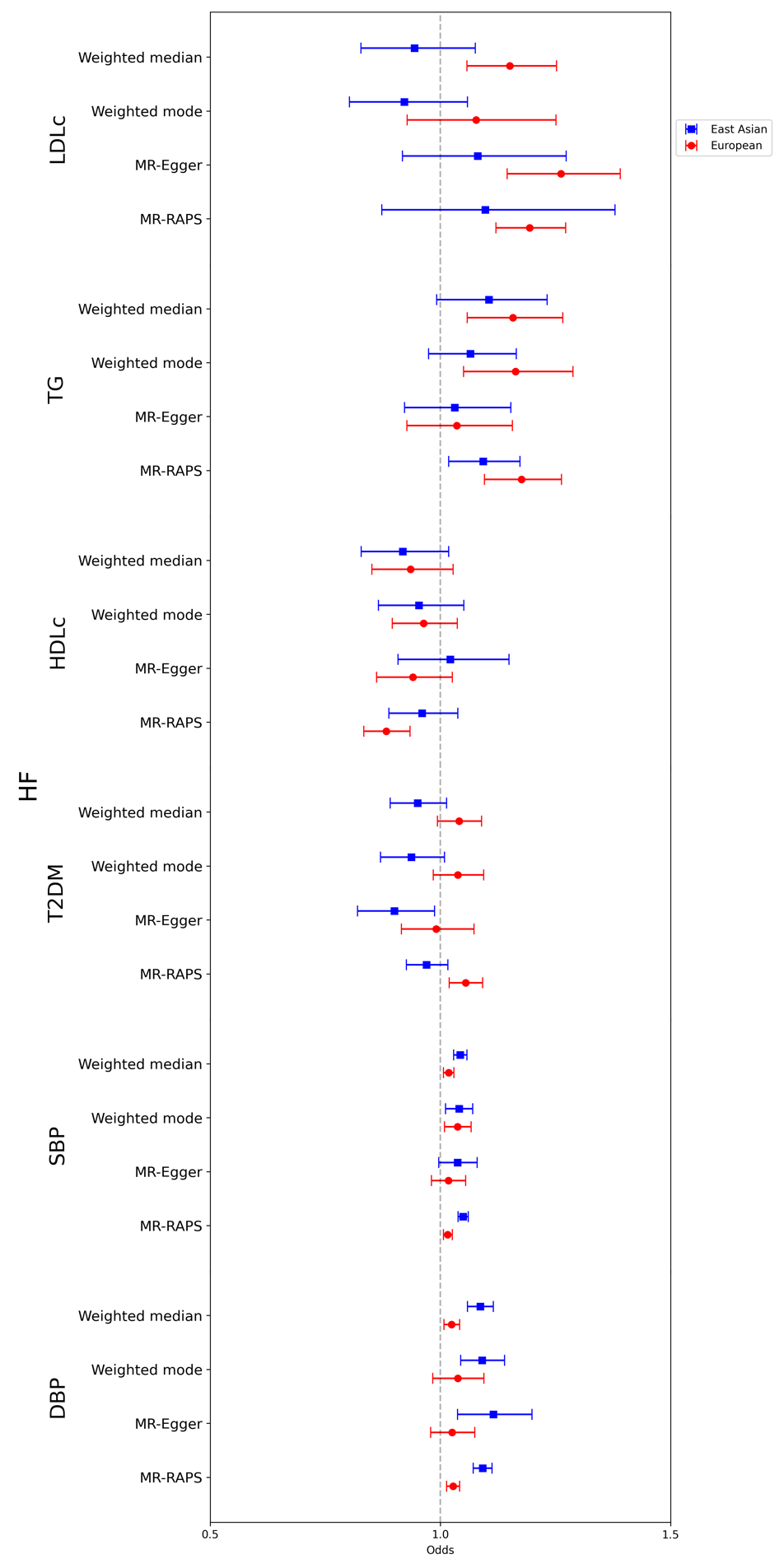


**E**


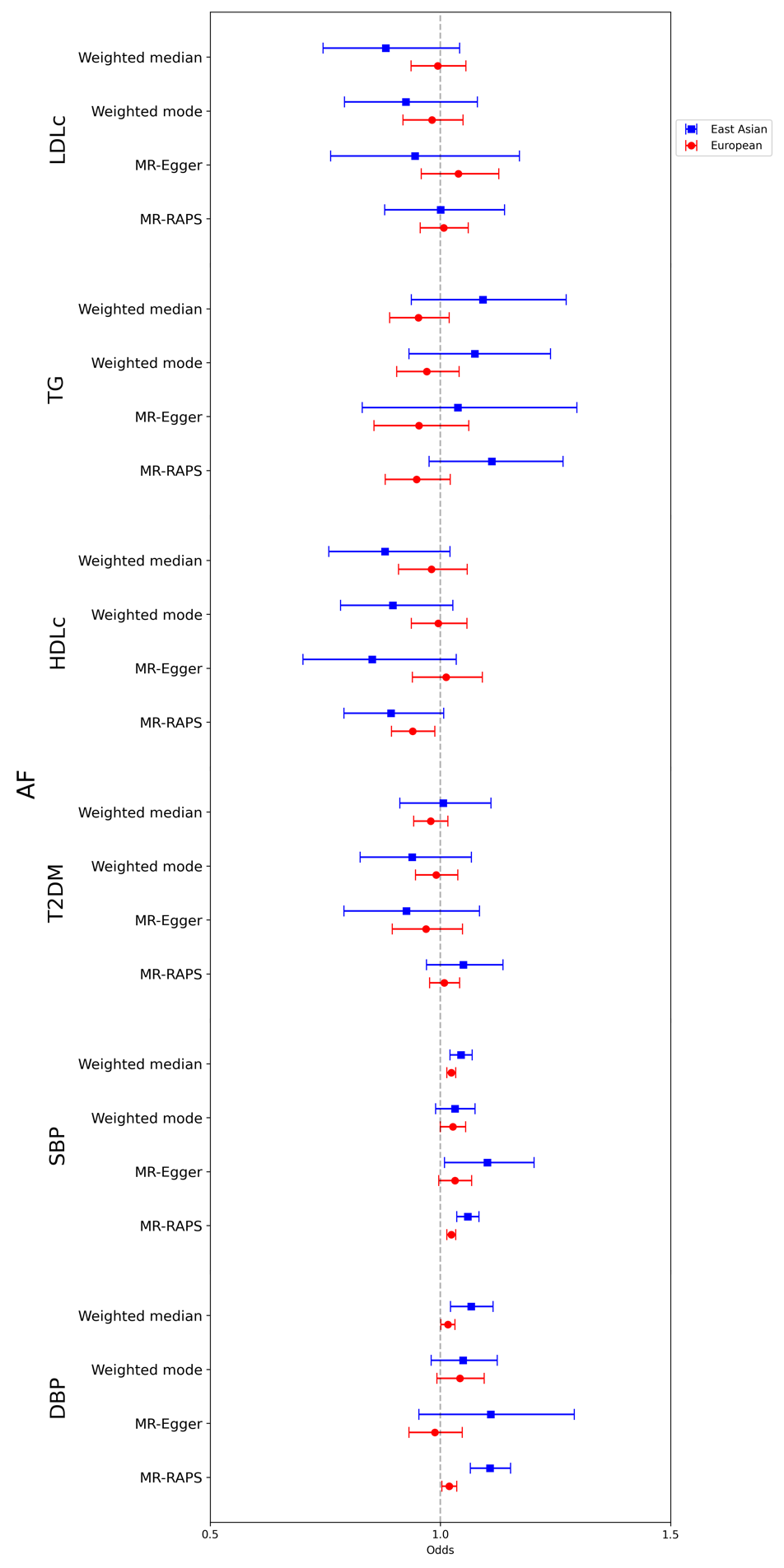


Supplementary Figure 1: Sensitivity analyses for the effect of one standard deviation increase in genetically predicted elevated low-density lipoprotein cholesterol (LDL-c), triglycerides (TG) and high-density lipoprotein cholesterol (HDL-c), per mmHg increase in systolic blood pressure (SBP), diastolic blood pressure (DBP) and type 2 diabetes mellitus liability (T2DM) on risk of ischemic heart disease (**A**), ischemic stroke (**B**), peripheral vascular disease (**C**), heart failure (**D**) and atrial fibrillation (**E**).


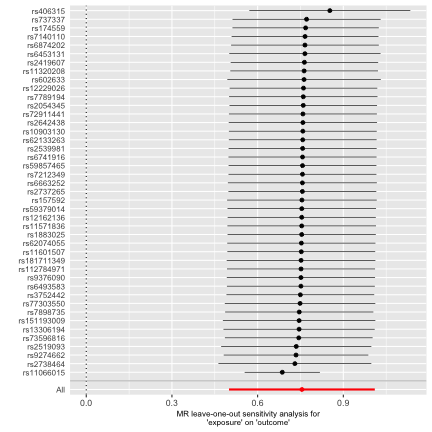


**i**


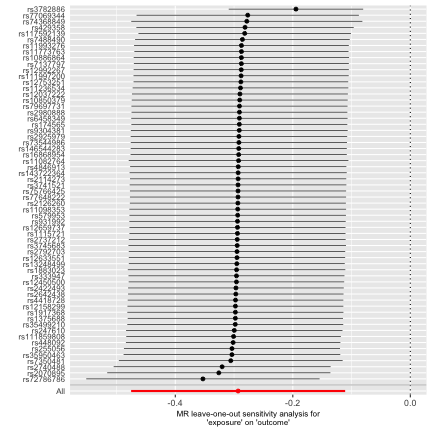


**ii**


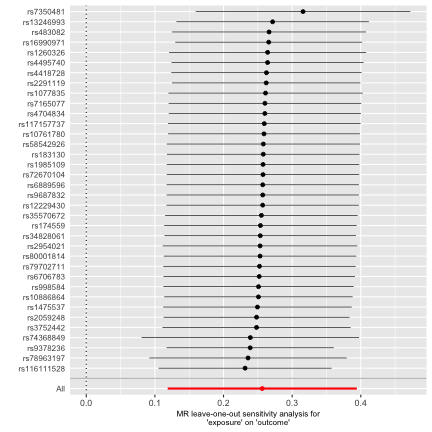


**iii**


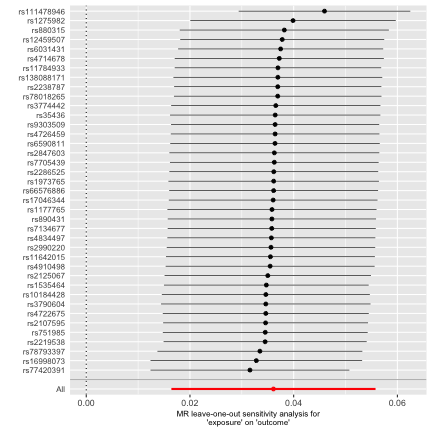


**iv**


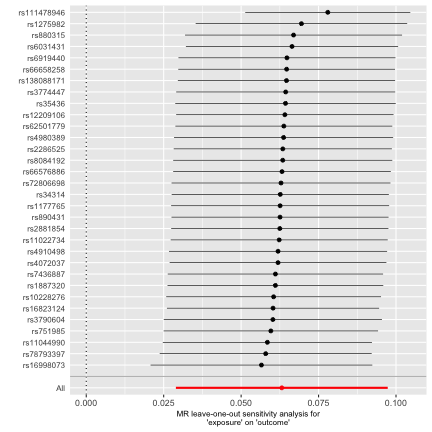


**v**


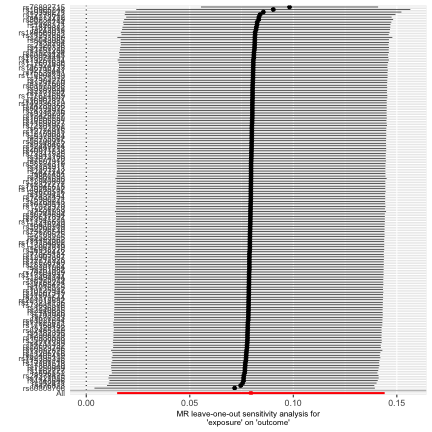


**vi**


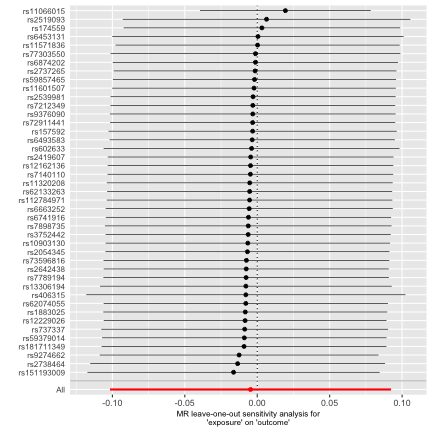


**vii**


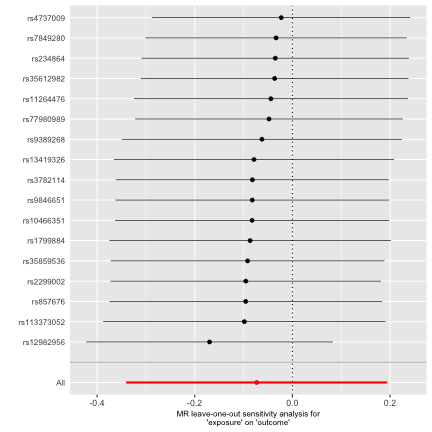


**viii**


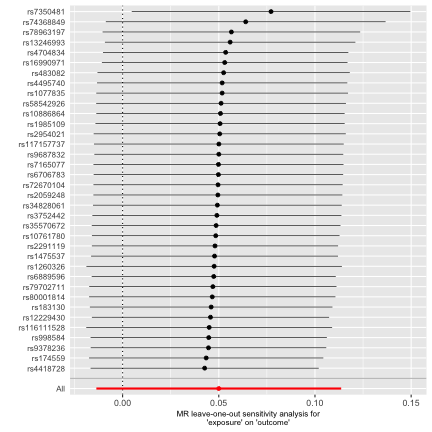


**ix**


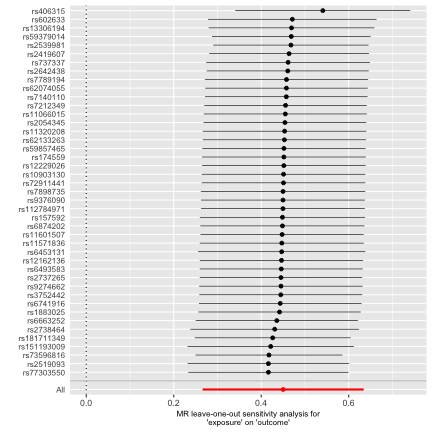


**xiii**


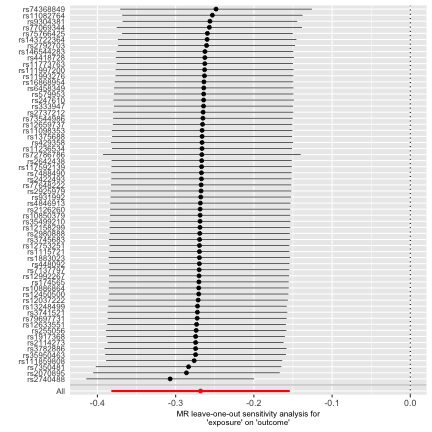


**xiv**


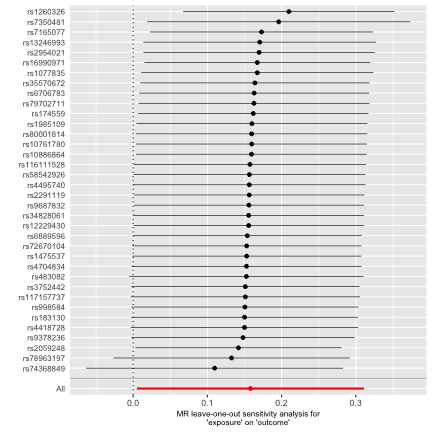


**xv**


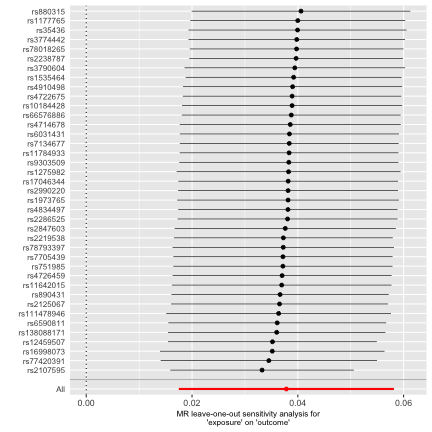


**xvi**


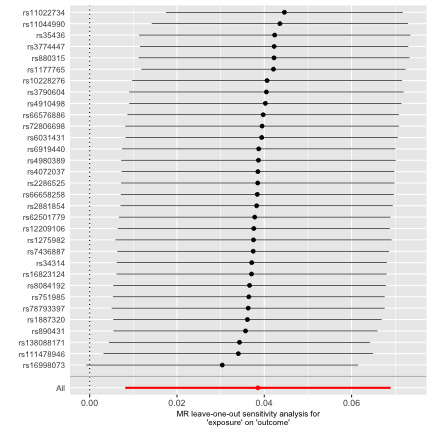


**xvii**


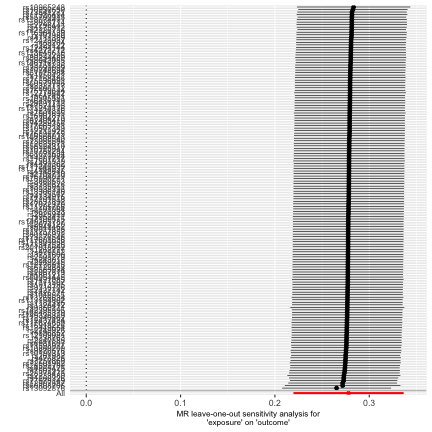


**xviii**


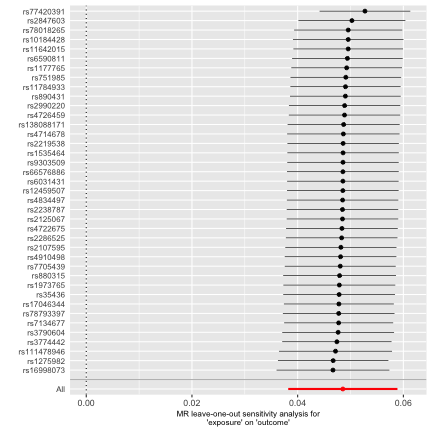


**x**


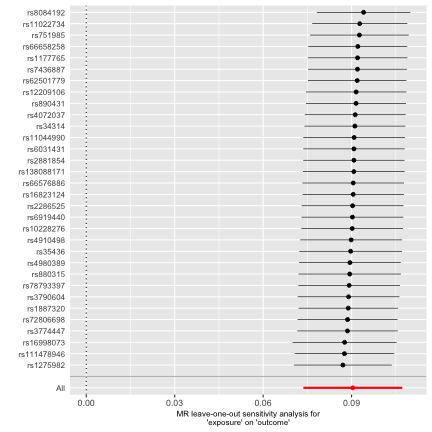


**xi**


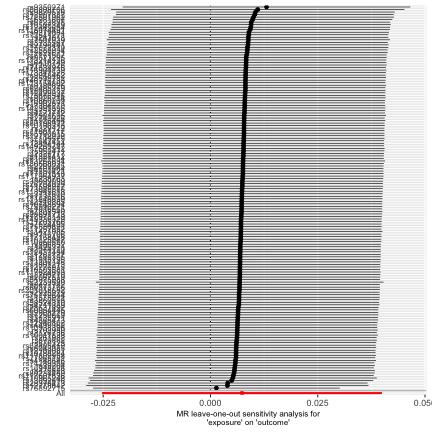


**xii**


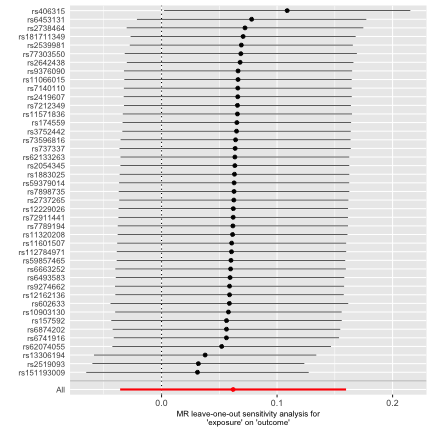


**xix**


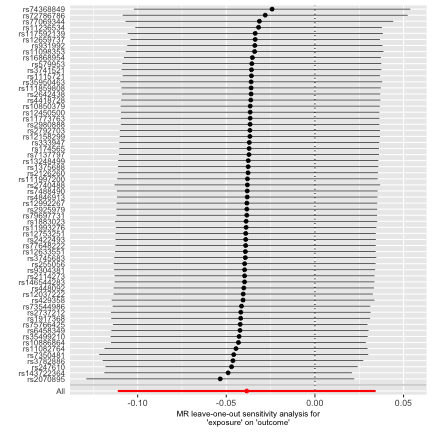


**xx**


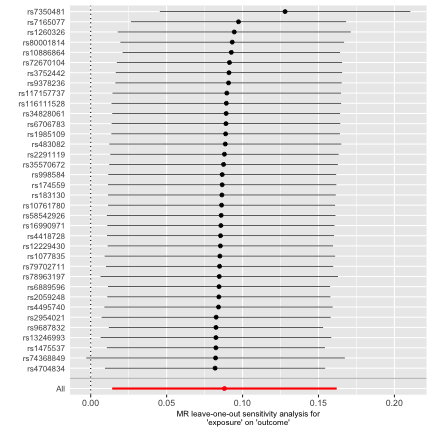


**xxi**


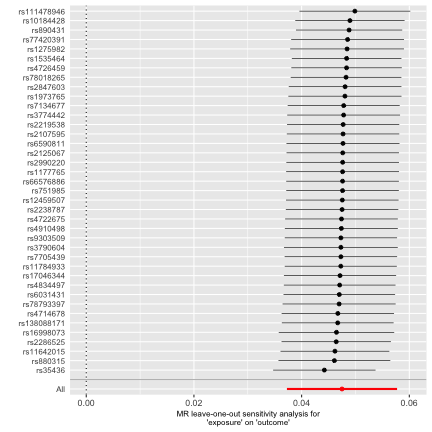


**xxii**


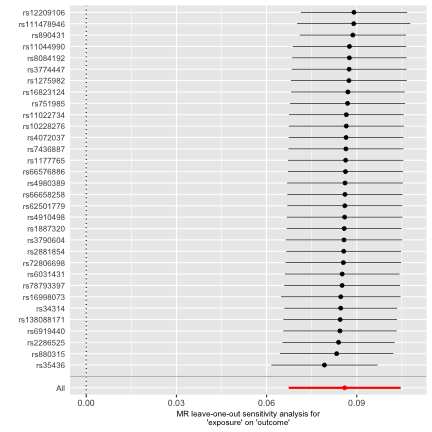


**xxiii**


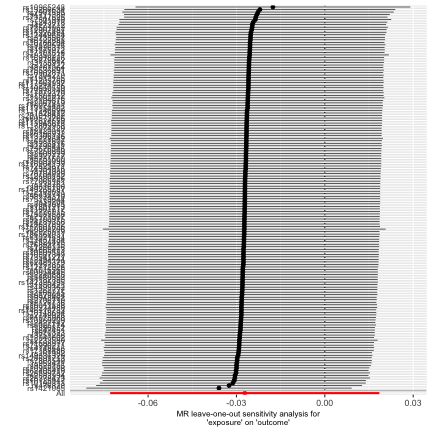


**xxiv**


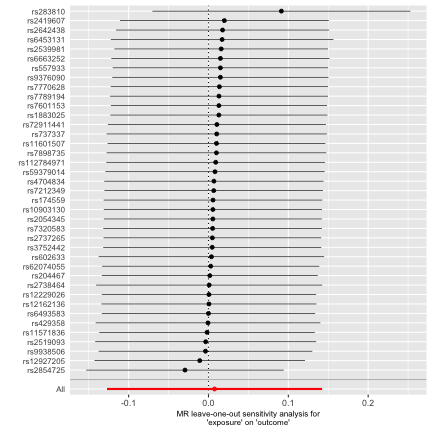


**xxv**


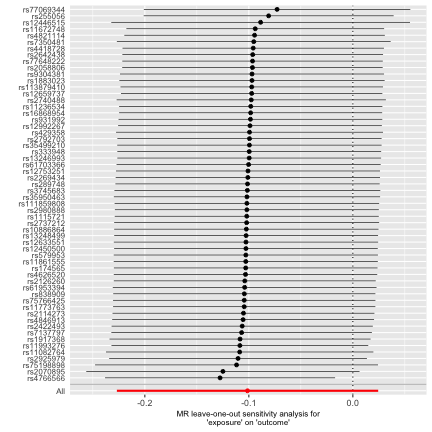


**xxvi**


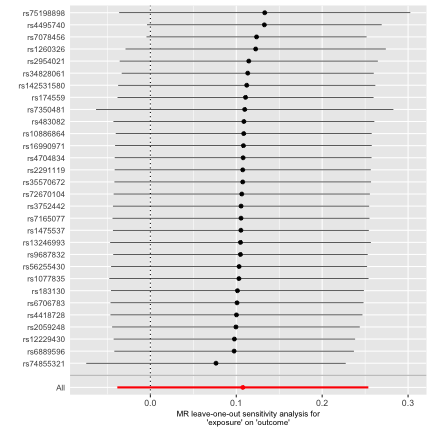


**xxvii**


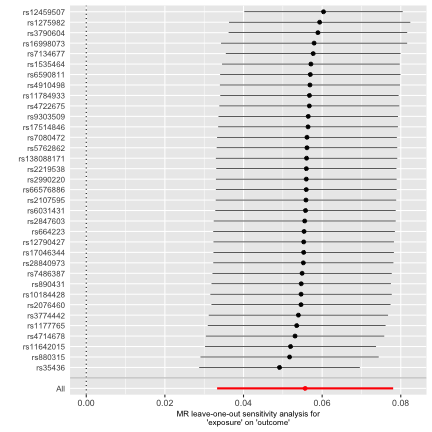


**xxviii**


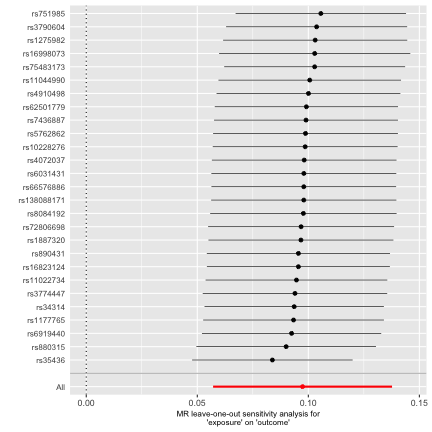


**xxix**


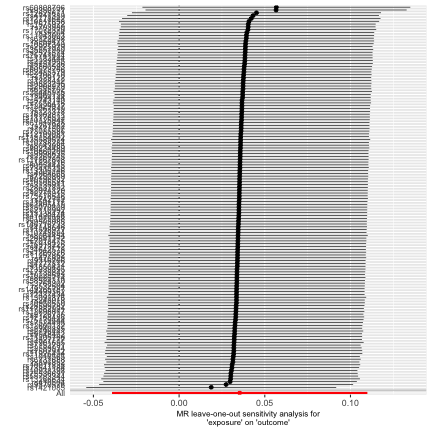


**xxx**

Supplementary Figure 2. Leave-one-out analyses for each exposure x outcome pair from the East Asian analysis. **(i)** LDL-c x IHD. **(ii)** HDL-c x IHD. **(iii)** TG x IHD. **(iv)** SBP x IHD. **(v)** DBP x IHD. **(vi)** T2DM x IHD. **(vii)** LDL-c x IS. **(viii)** HDL-c x IS. **(ix)** TG x IS. **(x)** SBP x IS. **(xi)** DBP x IS. **(xii)** T2DM x IS. **(xiii)** LDL-c x PVD. **(xiv)** HDL-c x PVD. **(xv)** TG x PVD. **(xvi)** SBP x PVD. **(xvii)** DBP x PVD. **(xviii)** T2DM x PVD. **(xix)** LDL-c x HF. **(xx)** HDL-c x HF. **(xxi)** TG x HF. **(xxii)** SBP x HF. **(xxiii)** DBP x HF. **(xxiv)** T2DM x HF.  **(xxv)**

LDL-c x AF. **(xxvi)** HDL-c x AF. **(xxvii)** TG x AF. **(xxviii)** SBP x AF. **(xxix)** DBP x AF. **(xxx)** T2DM x AF. LDL-c: low-density lipoprotein cholesterol; TG: triglycerides; HDL-c: high-density lipoprotein cholesterol; SBP: systolic blood pressure; DBP: diastolic blood pressure; T2DM: type 2 diabetes mellitus; IHD: ischemic heart disease; IS: ischemic stroke; PVD: peripheral vascular disease; HF: heart failure; AF: atrial fibrillation.


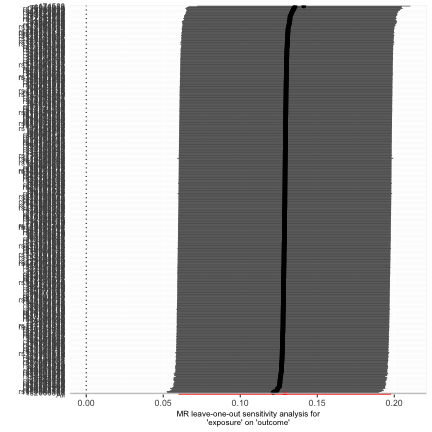

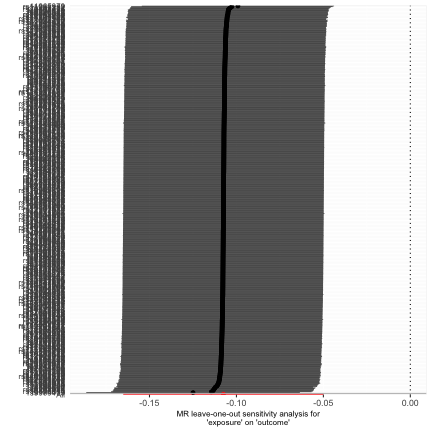

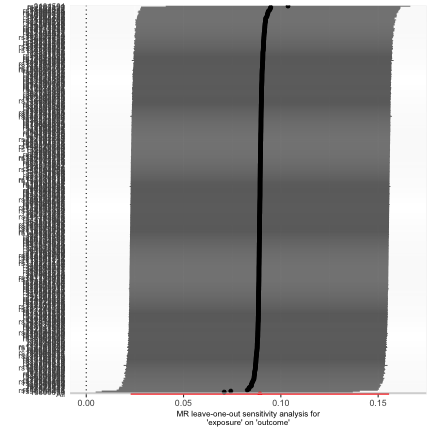

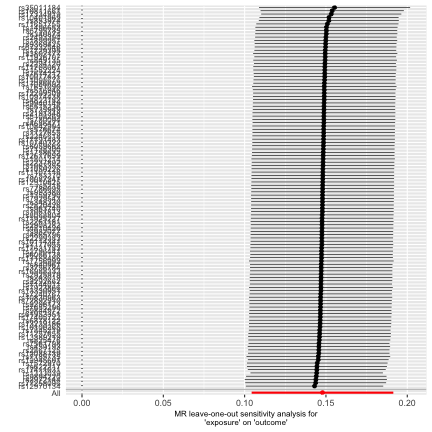

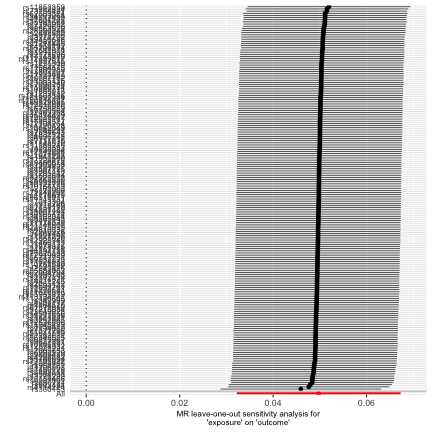

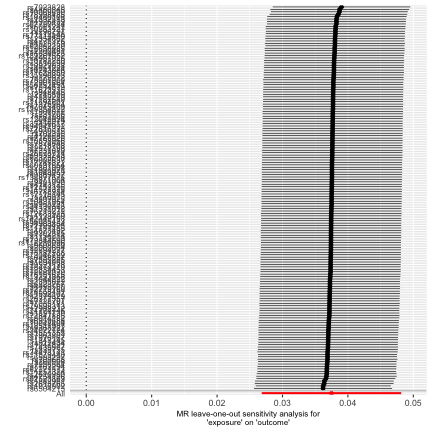

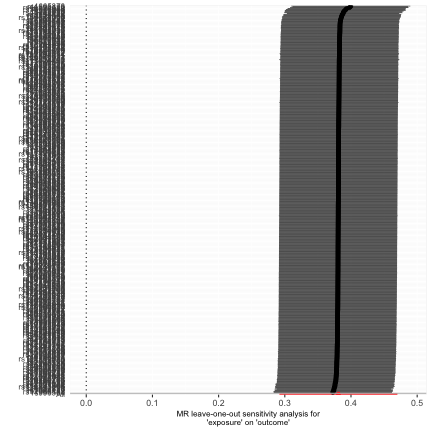

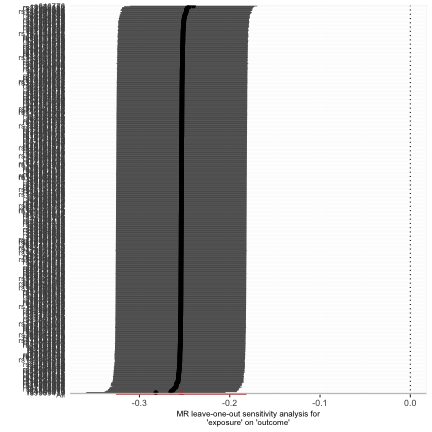


**i**

**ii**

**iii**

**iv**

**v**

**vi**

**vii**

**viii**

**ix**


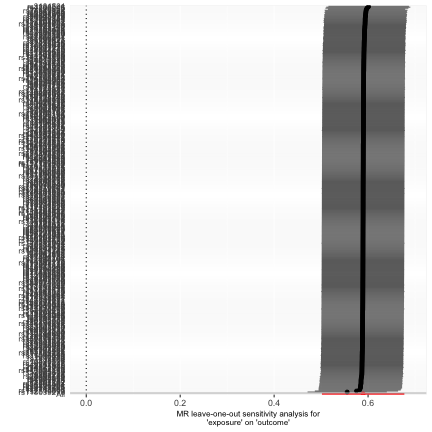

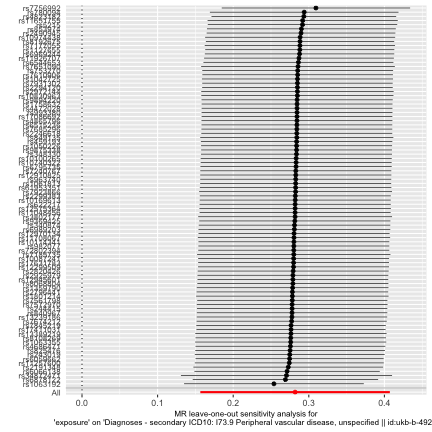

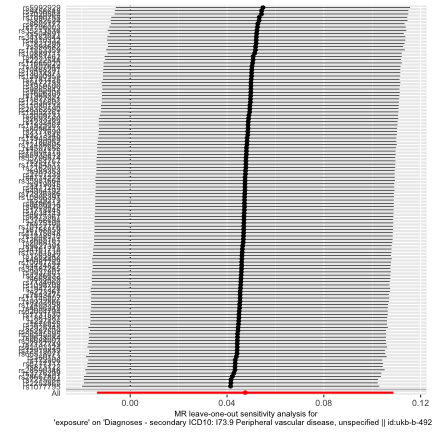

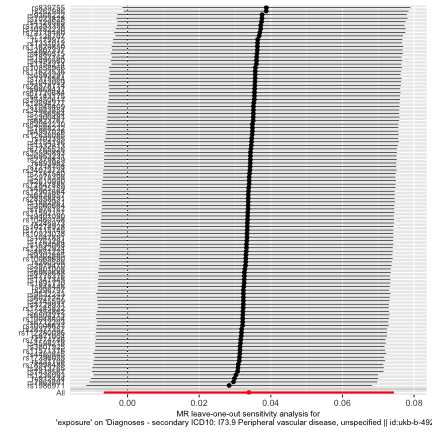

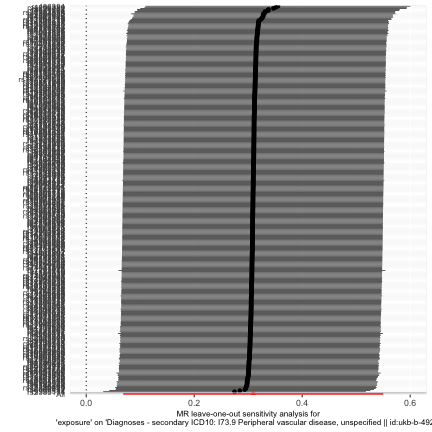

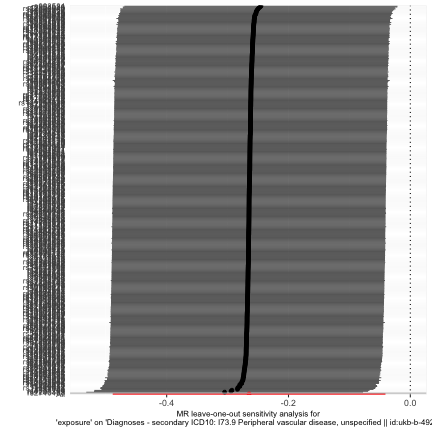

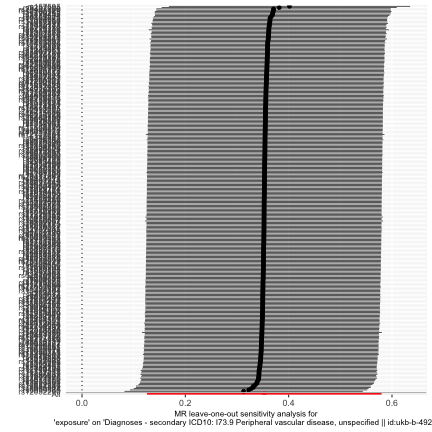

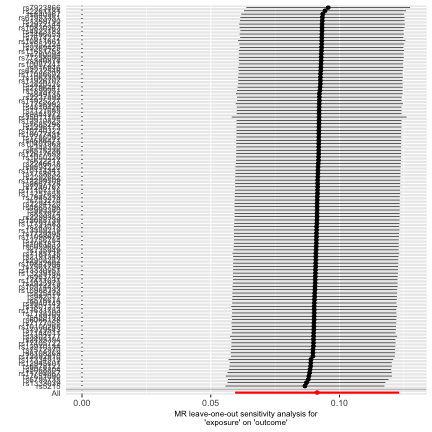

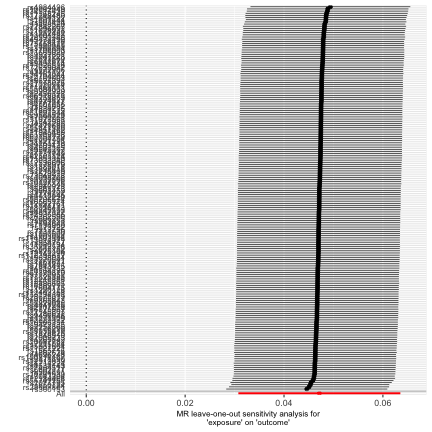

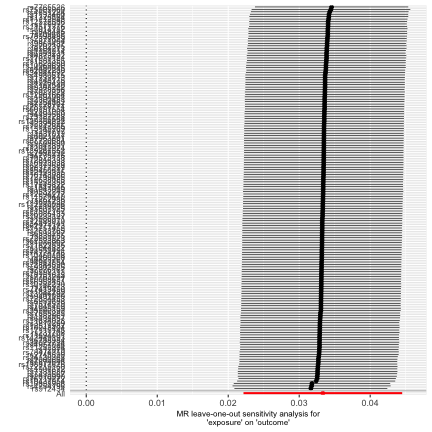


**xiii**

**xiv**

**xv**

**xvi**

**xvii**

**xviii**

**x**

**xi**

**xii**


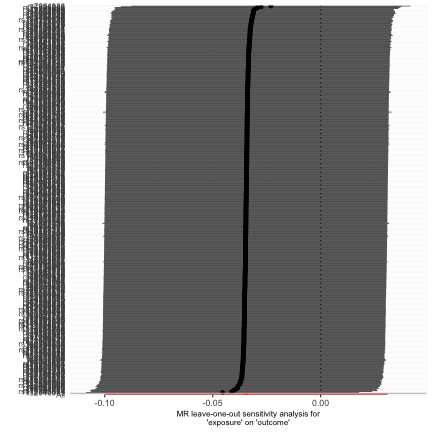

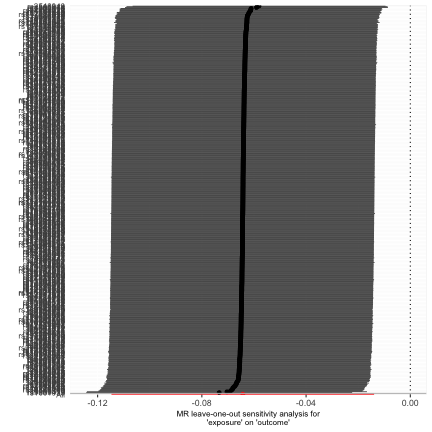

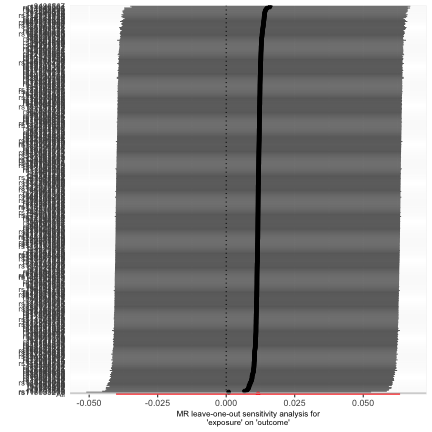

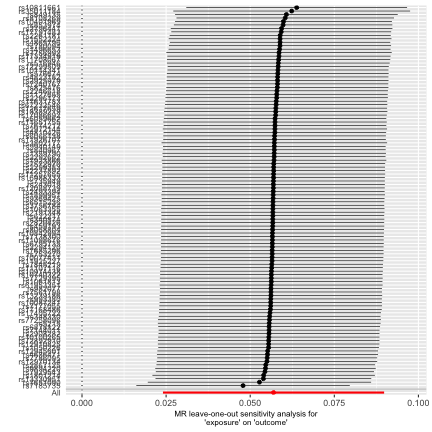

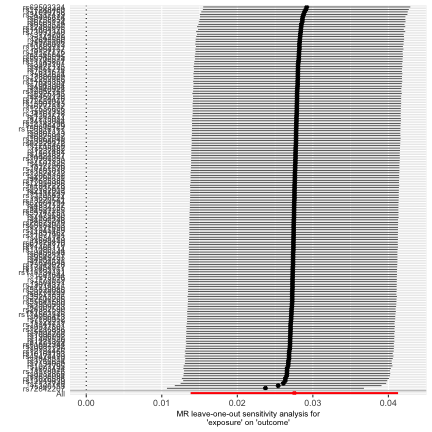

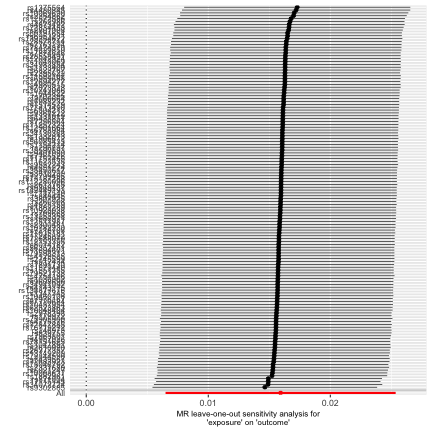

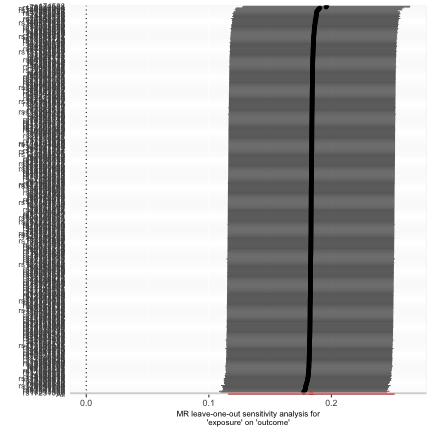

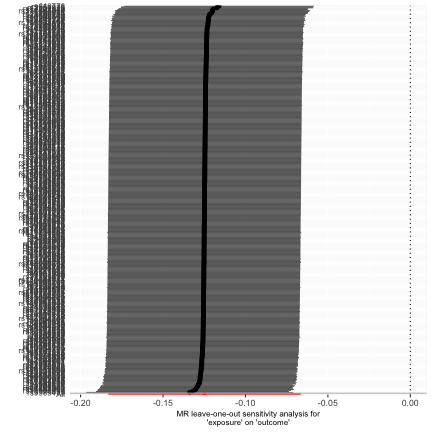

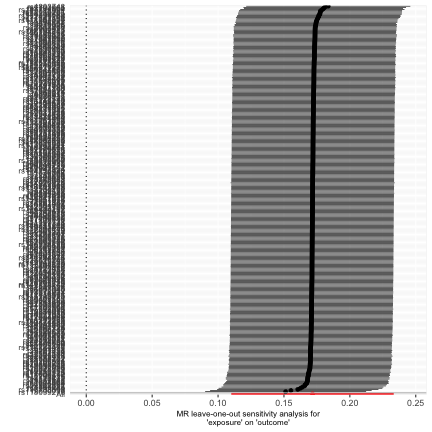


**xix**

**xx**

**xxi**

**xxii**

**xxiii**

**xxiv**

**xxv**

**xxvi**

**xxvii**


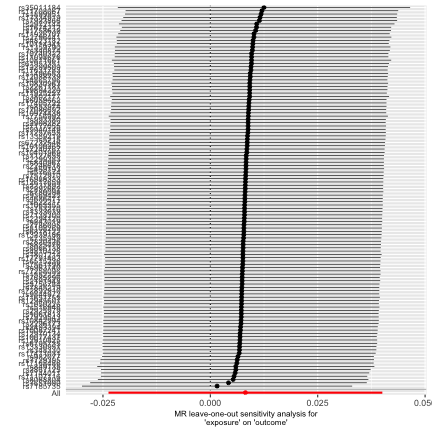

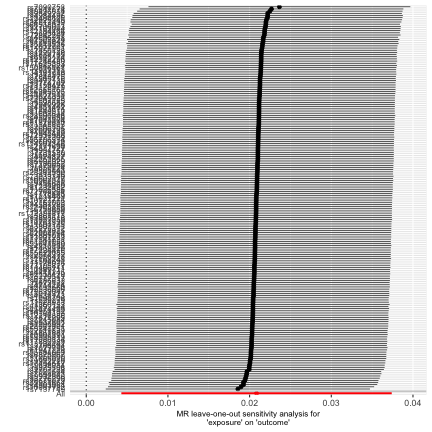

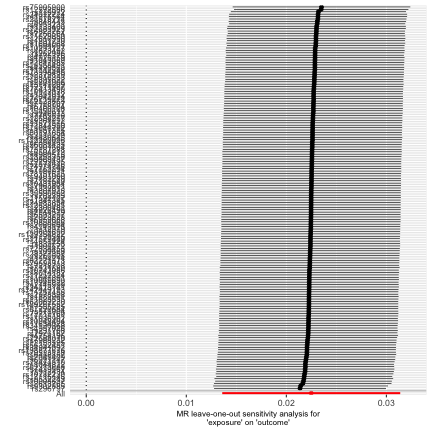


**xxviii**

**xxix**

**xxx**

Supplementary Figure 3. Leave-one-out analyses for each exposure x outcome pair from the European analysis. **(i)** LDL-c x IHD. **(ii)** HDL-c x IHD. **(iii)** TG x IHD. **(iv)** SBP x IHD. **(v)** DBP x IHD. **(vi)** T2DM x IHD. **(vii)** LDL-c x IS. **(viii)** HDL-c x IS. **(ix)** TG x IS. **(x)** SBP x IS. **(xi)** DBP x IS. **(xii)** T2DM x IS. **(xiii)** LDL-c x PVD. **(xiv)** HDL-c x PVD. **(xv)** TG x PVD. **(xvi)** SBP x PVD. **(xvii)** DBP x PVD. **(xviii)** T2DM x PVD. **(xix)** LDL-c x HF. **(xx)** HDL-c x HF. **(xxi)** TG x HF. **(xxii)** SBP x HF. **(xxiii)** DBP x HF. **(xxiv)** T2DM x HF.  **(xxv)**

LDL-c x AF. **(xxvi)** HDL-c x AF. **(xxvii)** TG x AF. **(xxviii)** SBP x AF. **(xxix)** DBP x AF. **(xxx)** T2DM x AF. LDL-c: low-density lipoprotein cholesterol; TG: triglycerides; HDL-c: high-density lipoprotein cholesterol; SBP: systolic blood pressure; DBP: diastolic blood pressure; T2DM: type 2 diabetes mellitus; IHD: ischemic heart disease; IS: ischemic stroke; PVD: peripheral vascular disease; HF: heart failure; AF: atrial fibrillation.


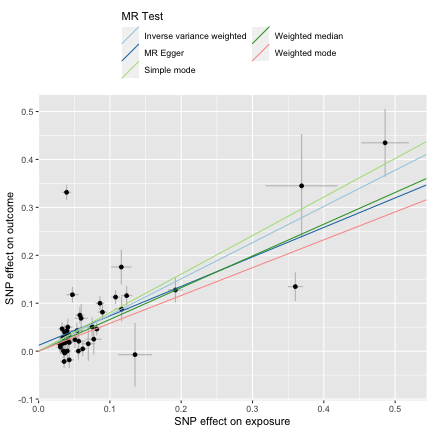


**i**


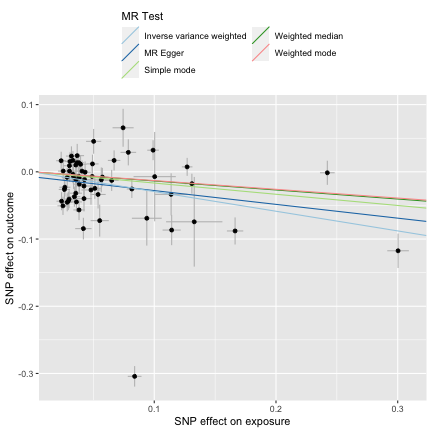


**ii**


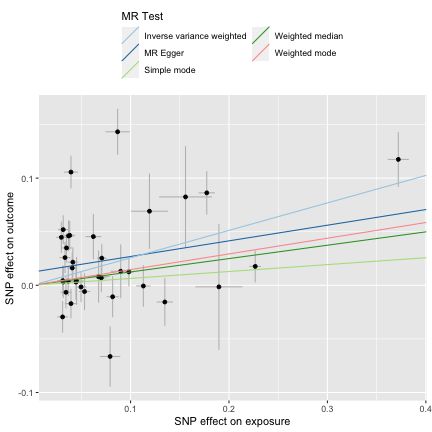

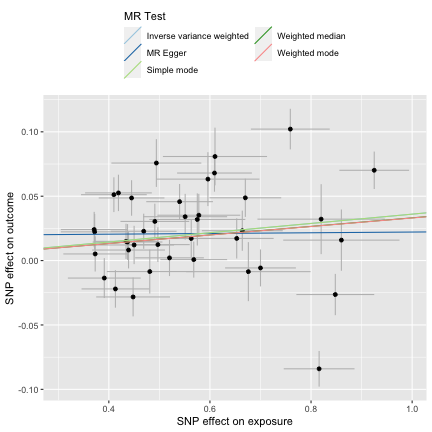


**iv**


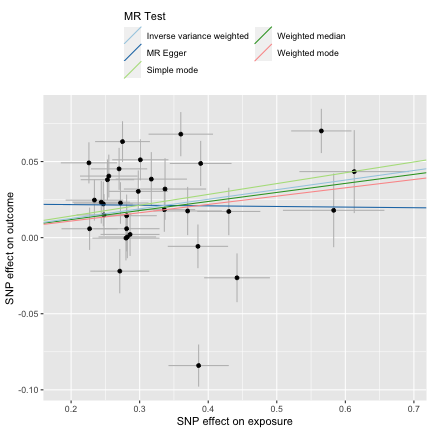


**v**


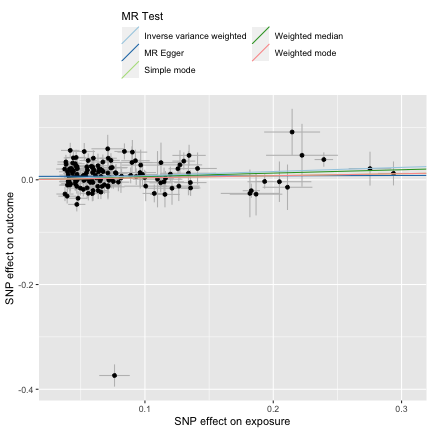


**vi**

**iii**


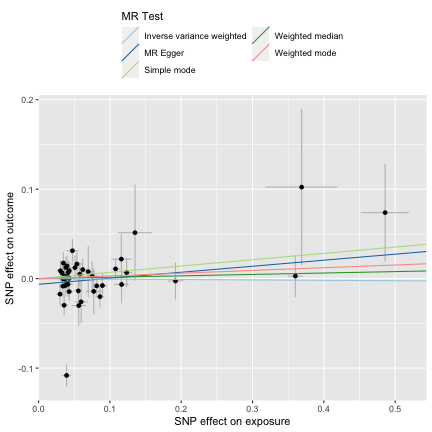


**vii**


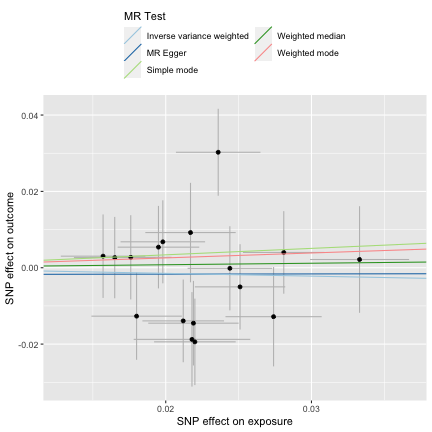


**viii**


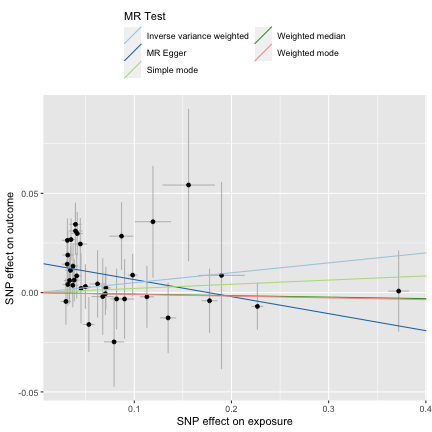


**ix**


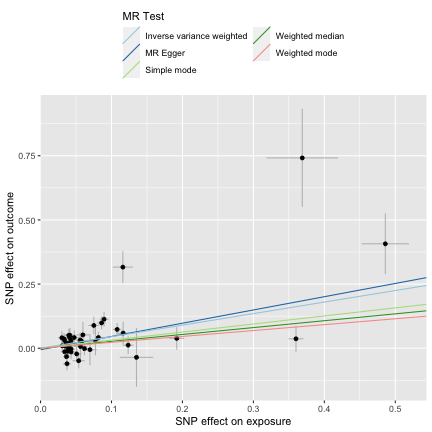


**xiii**


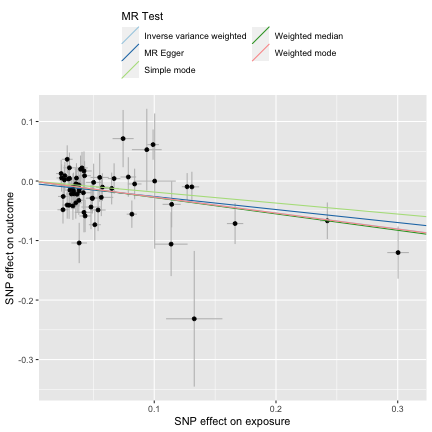


**xiv**


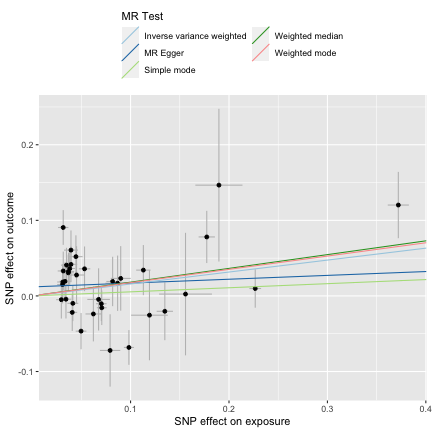


**xv**


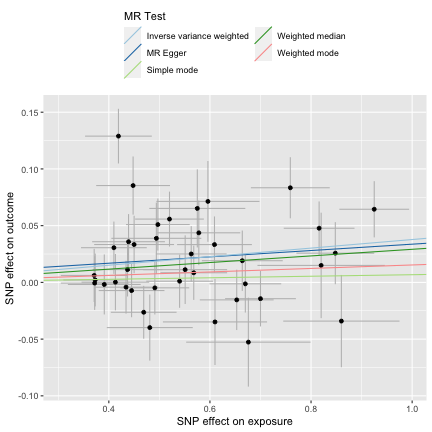


**xvi**


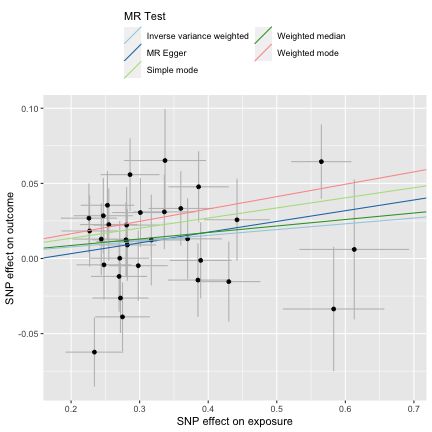


**xvii**


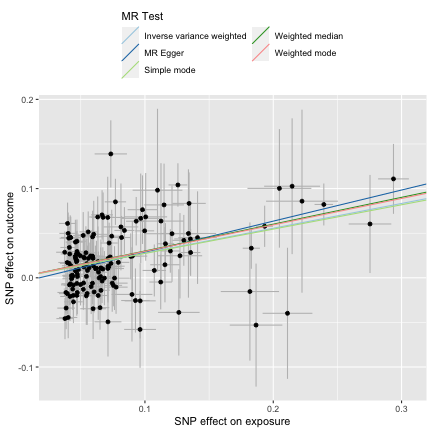


**xviii**


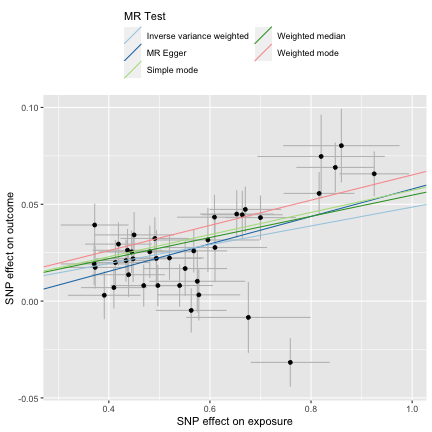


**x**


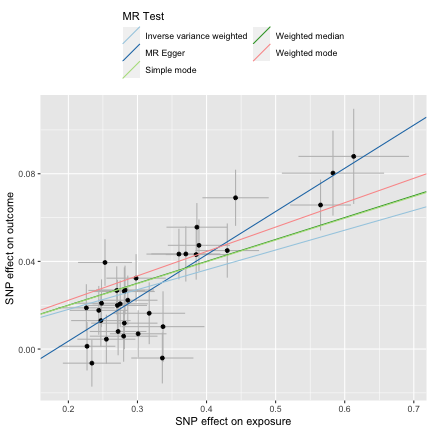


**xi**


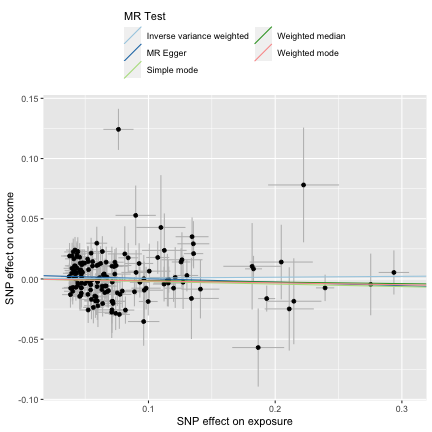


**xii**


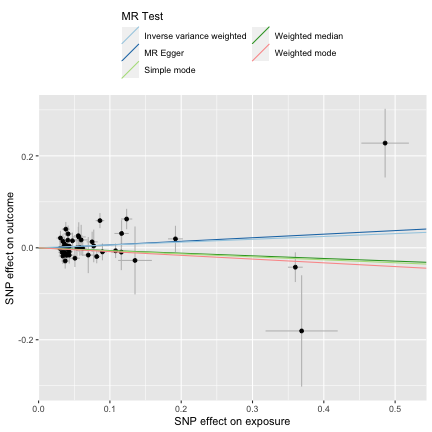


**xix**


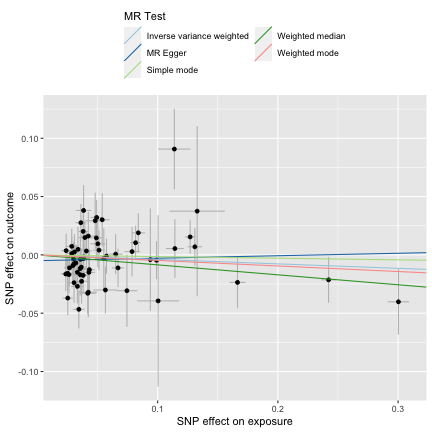


**xx**


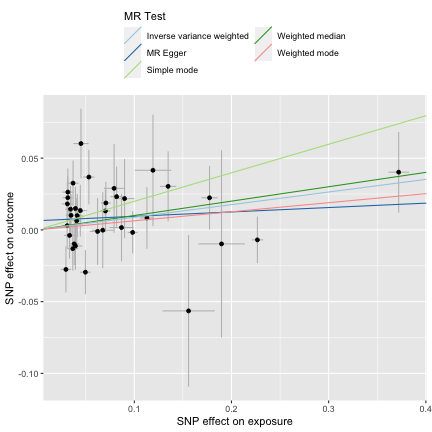


**xxi**


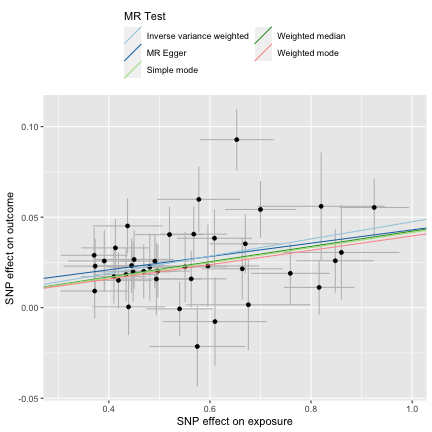


**xxii**


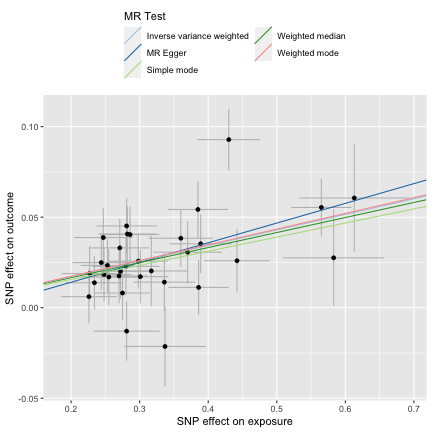


**xxiii**


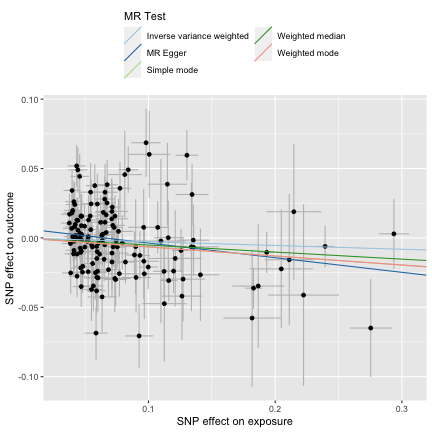


**xxiv**


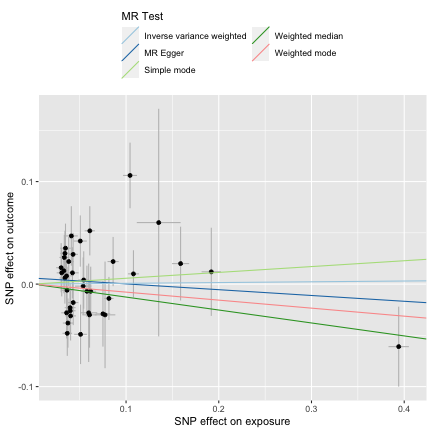


**xxv**


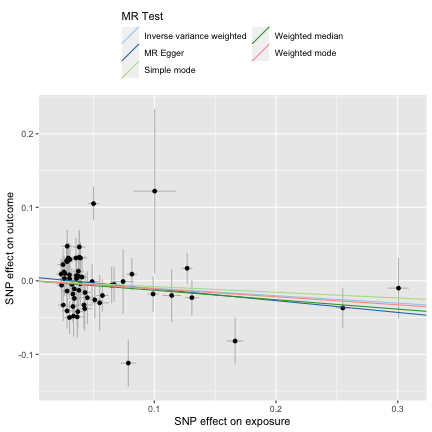


**xxvi**


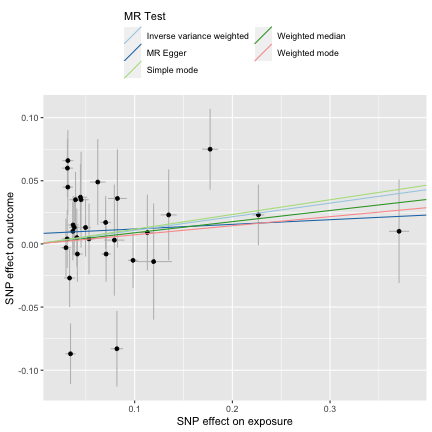


**xxvii**


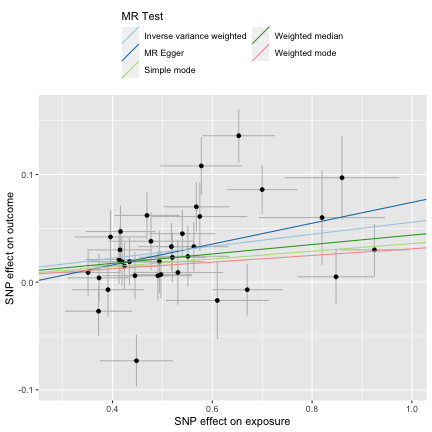


**xxviii**


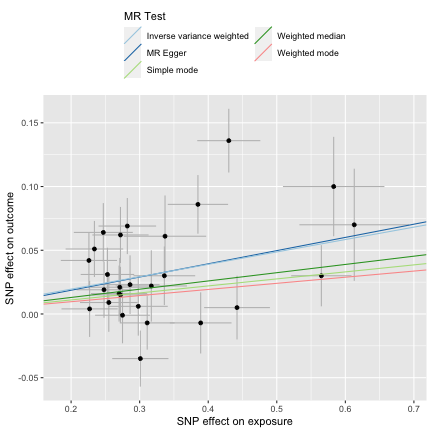


**xxix**


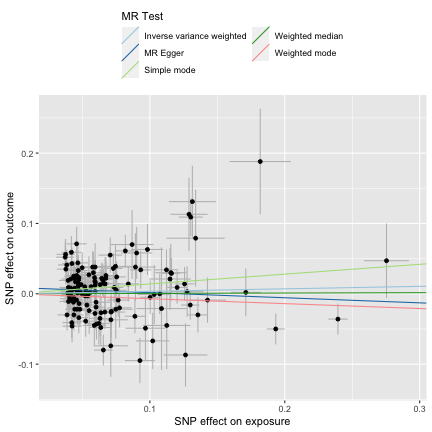


**xxx**

Supplementary Figure 4. Scatter plots for each exposure x outcome pair from the East Asian analyses. **(i)** LDL-c x IHD. **(ii)** HDL-c x IHD. **(iii)** TG x IHD. **(iv)** SBP x IHD. **(v)** DBP x IHD. **(vi)** T2DM x IHD. **(vii)** LDL-c x IS. **(viii)** HDL-c x IS. **(ix)** TG x IS. **(x)** SBP x IS. **(xi)** DBP x IS. **(xii)** T2DM x IS. **(xiii)** LDL-c x PVD. **(xiv)** HDL-c x PVD. **(xv)** TG x PVD. **(xvi)** SBP x PVD. **(xvii)** DBP x PVD. **(xviii)** T2DM x PVD. **(xix)** LDL-c x HF. **(xx)** HDL-c x HF. **(xxi)** TG x HF. **(xxii)** SBP x HF. **(xxiii)** DBP x HF. **(xxiv)** T2DM x HF.  **(xxv)**

LDL-c x AF. **(xxvi)** HDL-c x AF. **(xxvii)** TG x AF. **(xxviii)** SBP x AF. **(xxix)** DBP x AF. **(xxx)** T2DM x AF. LDL-c: low-density lipoprotein cholesterol; TG: triglycerides; HDL-c: high-density lipoprotein cholesterol; SBP: systolic blood pressure; DBP: diastolic blood pressure; T2DM: type 2 diabetes mellitus; IHD: ischemic heart disease; IS: ischemic stroke; PVD: peripheral vascular disease; HF: heart failure; AF: atrial fibrillation.


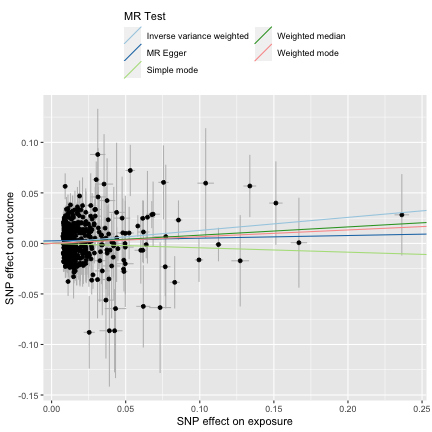

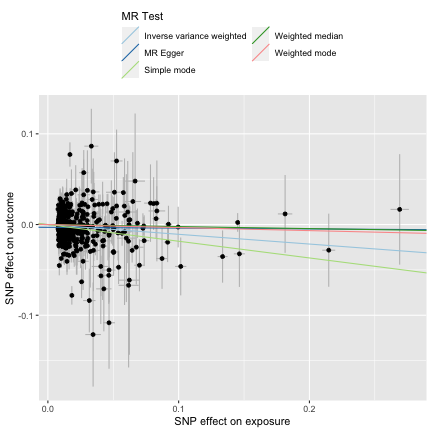

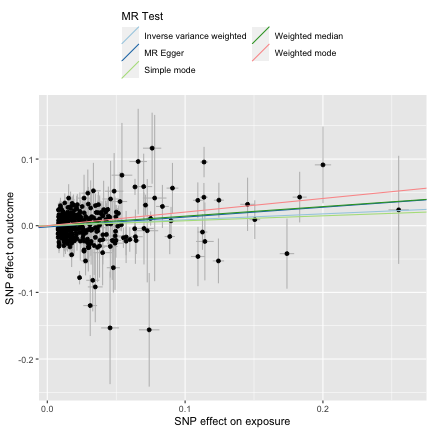

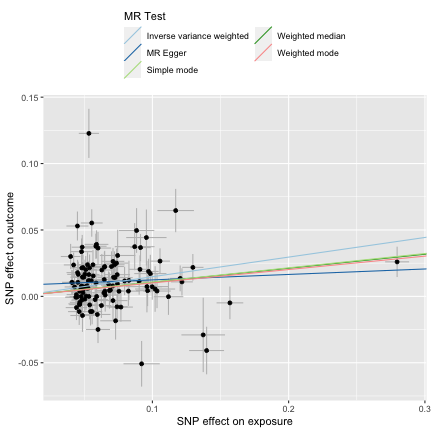

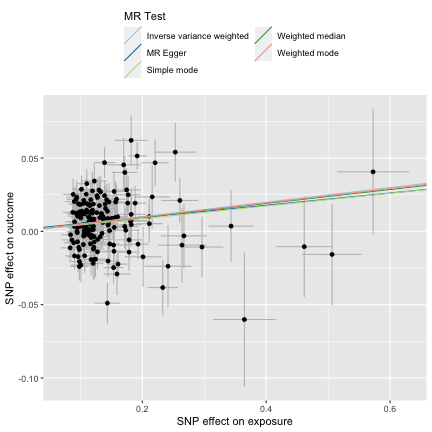


**i**

**ii**

**iv**

**v**

**vi**

**iii**

**vii**

**viii**

**ix**

**xiii**

**xiv**

**xv**

**xvi**

**xvii**

**xviii**

**x**

**xi**

**xii**

**xix**

**xx**

**xxi**

**xxii**

**xxiii**

**xxiv**

**xxv**

**xxvi**

**xxvii**

**xxviii**

**xxix**

**xxx**

Supplementary Figure 5. Scatter plots for each exposure x outcome pair from the European analyses. **(i)** LDL-c x IHD. **(ii)** HDL-c x IHD. **(iii)** TG x IHD. **(iv)** SBP x IHD. **(v)** DBP x IHD. **(vi)** T2DM x IHD. **(vii)** LDL-c x IS. **(viii)** HDL-c x IS. **(ix)** TG x IS. **(x)** SBP x IS. **(xi)** DBP x IS. **(xii)** T2DM x IS. **(xiii)** LDL-c x PVD. **(xiv)** HDL-c x PVD. **(xv)** TG x PVD. **(xvi)** SBP x PVD. **(xvii)** DBP x PVD. **(xviii)** T2DM x PVD. **(xix)** LDL-c x HF. **(xx)** HDL-c x HF. **(xxi)** TG x HF. **(xxii)** SBP x HF. **(xxiii)** DBP x HF. **(xxiv)** T2DM x HF.  **(xxv)**

LDL-c x AF. **(xxvi)** HDL-c x AF. **(xxvii)** TG x AF. **(xxviii)** SBP x AF. **(xxix)** DBP x AF. **(xxx)** T2DM x AF. LDL-c: low-density lipoprotein cholesterol; TG: triglycerides; HDL-c: high-density lipoprotein cholesterol; SBP: systolic blood pressure; DBP: diastolic blood pressure; T2DM: type 2 diabetes mellitus; IHD: ischemic heart disease; IS: ischemic stroke; PVD: peripheral vascular disease; HF: heart failure; AF: atrial fibrillation.

**i**

**ii**

**iii**

**iv**

**v**

**vi**

**vii**

**viii**

**ix**

**xiii**

**xiv**

**xv**

**xvi**

**xvii**

**xviii**

**x**

**xi**

**xii**

**xix**

**xx**

**xxi**

**xxii**

**xxiii**

**xxiv**

**xxv**

**xxvi**

**xxvii**

**xxviii**

**xxix**

**xxx**

Supplementary Figure 6. Funnel plots for each exposure x outcome pair from the East Asian analyses. **(i)** LDL-c x IHD. **(ii)** HDL-c x IHD. **(iii)** TG x IHD. **(iv)** SBP x IHD. **(v)** DBP x IHD. **(vi)** T2DM x IHD. **(vii)** LDL-c x IS. **(viii)** HDL-c x IS. **(ix)** TG x IS. **(x)** SBP x IS. **(xi)** DBP x IS. **(xii)** T2DM x IS. **(xiii)** LDL-c x PVD. **(xiv)** HDL-c x PVD. **(xv)** TG x PVD. **(xvi)** SBP x PVD. **(xvii)** DBP x PVD. **(xviii)** T2DM x PVD. **(xix)** LDL-c x HF. **(xx)** HDL-c x HF. **(xxi)** TG x HF. **(xxii)** SBP x HF. **(xxiii)** DBP x HF. **(xxiv)** T2DM x HF.  **(xxv)**

LDL-c x AF. **(xxvi)** HDL-c x AF. **(xxvii)** TG x AF. **(xxviii)** SBP x AF. **(xxix)** DBP x AF. **(xxx)** T2DM x AF. LDL-c: low-density lipoprotein cholesterol; TG: triglycerides; HDL-c: high-density lipoprotein cholesterol; SBP: systolic blood pressure; DBP: diastolic blood pressure; T2DM: type 2 diabetes mellitus; IHD: ischemic heart disease; IS: ischemic stroke; PVD: peripheral vascular disease; HF: heart failure; AF: atrial fibrillation.

**i**

**ii**

**iii**

**iv**

**v**

**vi**

**vii**

**viii**

**ix**

**xiii**

**xiv**

**xv**

**xvi**

**xvii**

**xviii**

**x**

**xi**

**xii**

**xix**

**xx**

**xxi**

**xxii**

**xxiii**

**xxiv**

**xxv**

**xxvi**

**xxvii**

**xxviii**

**xxix**

**xxx**

Supplementary Figure 7. Funnel plots for each exposure x outcome pair from the European analyses. **(i)** LDL-c x IHD. **(ii)** HDL-c x IHD. **(iii)** TG x IHD. **(iv)** SBP x IHD. **(v)** DBP x IHD. **(vi)** T2DM x IHD. **(vii)** LDL-c x IS. **(viii)** HDL-c x IS. **(ix)** TG x IS. **(x)** SBP x IS. **(xi)** DBP x IS. **(xii)** T2DM x IS. **(xiii)** LDL-c x PVD. **(xiv)** HDL-c x PVD. **(xv)** TG x PVD. **(xvi)** SBP x PVD. **(xvii)** DBP x PVD. **(xviii)** T2DM x PVD. **(xix)** LDL-c x HF. **(xx)** HDL-c x HF. **(xxi)** TG x HF. **(xxii)** SBP x HF. **(xxiii)** DBP x HF. **(xxiv)** T2DM x HF.  **(xxv)**

LDL-c x AF. **(xxvi)** HDL-c x AF. **(xxvii)** TG x AF. **(xxviii)** SBP x AF. **(xxix)** DBP x AF. **(xxx)** T2DM x AF. LDL-c: low-density lipoprotein cholesterol; TG: triglycerides; HDL-c: high-density lipoprotein cholesterol; SBP: systolic blood pressure; DBP: diastolic blood pressure; T2DM: type 2 diabetes mellitus; IHD: ischemic heart disease; IS: ischemic stroke; PVD: peripheral vascular disease; HF: heart failure; AF: atrial fibrillation.
